# Viruses traverse the human proteome through peptide interfaces that can be biomimetically leveraged for drug discovery

**DOI:** 10.1073/pnas.2308776121

**Published:** 2024-01-22

**Authors:** Laurène Meyniel-Schicklin, Jérôme Amaudrut, Pierre Mallinjoud, Fabrice Guillier, Philippe E. Mangeot, Laetitia Lines, Anne Aublin-Gex, Caroline Scholtes, Claire Punginelli, Stéphane Joly, Florence Vasseur, Evelyne Manet, Henri Gruffat, Thomas Henry, Farès Halitim, Jean-Laurent Paparin, Peter Machin, Raphaël Darteil, Diane Sampson, Ivan Mikaelian, Lydie Lane, Vincent Navratil, Marie-Pierre Golinelli-Cohen, Fabiola Terzi, Patrice André, Vincent Lotteau, Jacky Vonderscher, Eric C. Meldrum, Benoit de Chassey

**Affiliations:** ^a^ENYO Pharma, Lyon 69008, France; ^b^Inventiva, Daix 21121, France; ^c^Centre International de Recherche en Infectiologie, University Lyon, Inserm, U1111, Université Claude Bernard Lyon 1, CNRS, UMR5308, Ecole Normale Supérieure de Lyon, Lyon 69007, France; ^d^Université de Paris, INSERM U1151, CNRS UMR 8253, Institut Necker Enfants Malades, Département “Croissance et Signalisation”, Paris 75015, France; ^e^Université de Lyon, Université Claude Bernard Lyon 1, INSERM 1052, CNRS 5286, Centre Léon Bérard, Centre de recherche en cancérologie de Lyon, Lyon 69373, France; ^f^Computer and Laboratory Investigation of Proteins of Human Origin Group, Swiss Institute of Bioinformatics, Lausanne 1015, Switzerland; ^g^Pôle Rhône-Alpes de bioinformatique, Rhône-Alpes Bioinformatics Center, Université Lyon 1, Villeurbanne 69622, France; ^h^European Virus Bio-informatiques Center, Jena 07743, Germany; ^i^Institut Français de Bioinformatique, IFB-core, UMS 3601, Évry 91057, France; ^j^Université Paris-Saclay, CNRS, Institut de Chimie des Substances Naturelles, Unité Propre de Recherche 2301, Gif-sur-Yvette 91198, France

**Keywords:** interactomics, viruses, chemoinformatics, biomimetism, kidney

## Abstract

We present a drug design strategy based on structural knowledge of protein–protein interfaces selected through virus–host coevolution and translated into highly potential small molecules. This approach is grounded on Vinland, the most comprehensive atlas of virus–human protein–protein interactions with annotation of interacting domains. From this inspiration, we identified small viral protein domains responsible for interaction with human proteins. These peptides form a library of new chemical entities used to screen for replication modulators of several pathogens. As a proof of concept, a peptide from a KSHV protein, identified as an inhibitor of influenza virus replication, was translated into a small molecule series with low nanomolar antiviral activity. By targeting the NEET proteins, these molecules turn out to be of therapeutic interest in a nonalcoholic steatohepatitis mouse model with kidney lesions. This study provides a biomimetic framework to design original chemistries targeting cellular proteins, with indications going far beyond infectious diseases.

## Significance

Viruses have designed small protein interfaces to interact with human proteins. These viral peptides are original molecules to modulate the activity of host targets and an inspiration to create original drugs. Here, the wealth of virus–host protein interactions existing in the literature is integrated in an substantial database. A sample peptide library is screened against several pathogens, highlighting peptides modulators of replication. From one of them, a drug discovery program identifies highly potent antiviral molecules interacting with human metabolic targets. These molecules are proven to be active for treatment of mouse model of nonalcoholic steatohepatitis with chronic kidney disease. Our approach validates an original biomimetic framework to address cellular functions for fundamental applications and drug discovery.

After 60 y of constant decline (1), the trend line of the pharmaceutical industry R&D productivity started to change in the last decade (2), and the number of new molecular entities approved by the Food and Drug Administration (FDA) in 2020 was close to the highest total ever of 1996 (3). Nevertheless, the average cost to develop a single drug still exceeds 1 billion US Dollar and the attrition in the drug development pipeline has remained high (4). The identification of factors contributing to the recent positive turnaround signal in drug development is pivotal to strengthen productivity and contribute to innovation. One essential factor is the accumulation of knowledge through multiple omics approaches and a more optimal use of this information (2). In this context, we present an innovative systems biology-based approach that capitalizes on the knowledge of viruses and their unique properties acquired by evolution to identify original small molecular entities directed at attractive human disease targets.

Viruses are obligate intracellular parasites. Throughout their life cycle inside cells, their replication relies upon modulation of host cell functions. During their coevolution with their hosts, viruses became able to control a variety of biological processes through interaction with cellular proteins. Evolution has selected small viral protein interfaces that are necessary and sufficient to interact with host proteins (5, 6). Considerable work has been done to reveal the book of virus–host protein–protein interactions (vh-PPI) for a large range of viruses, including with the use of high-throughput technologies (7, 8). These data are scattered throughout the scientific literature with some attempts to integrate this information in databases, but not in an exhaustive manner and details on the interfaces responsible for interaction (9).

**Fig. 1.**
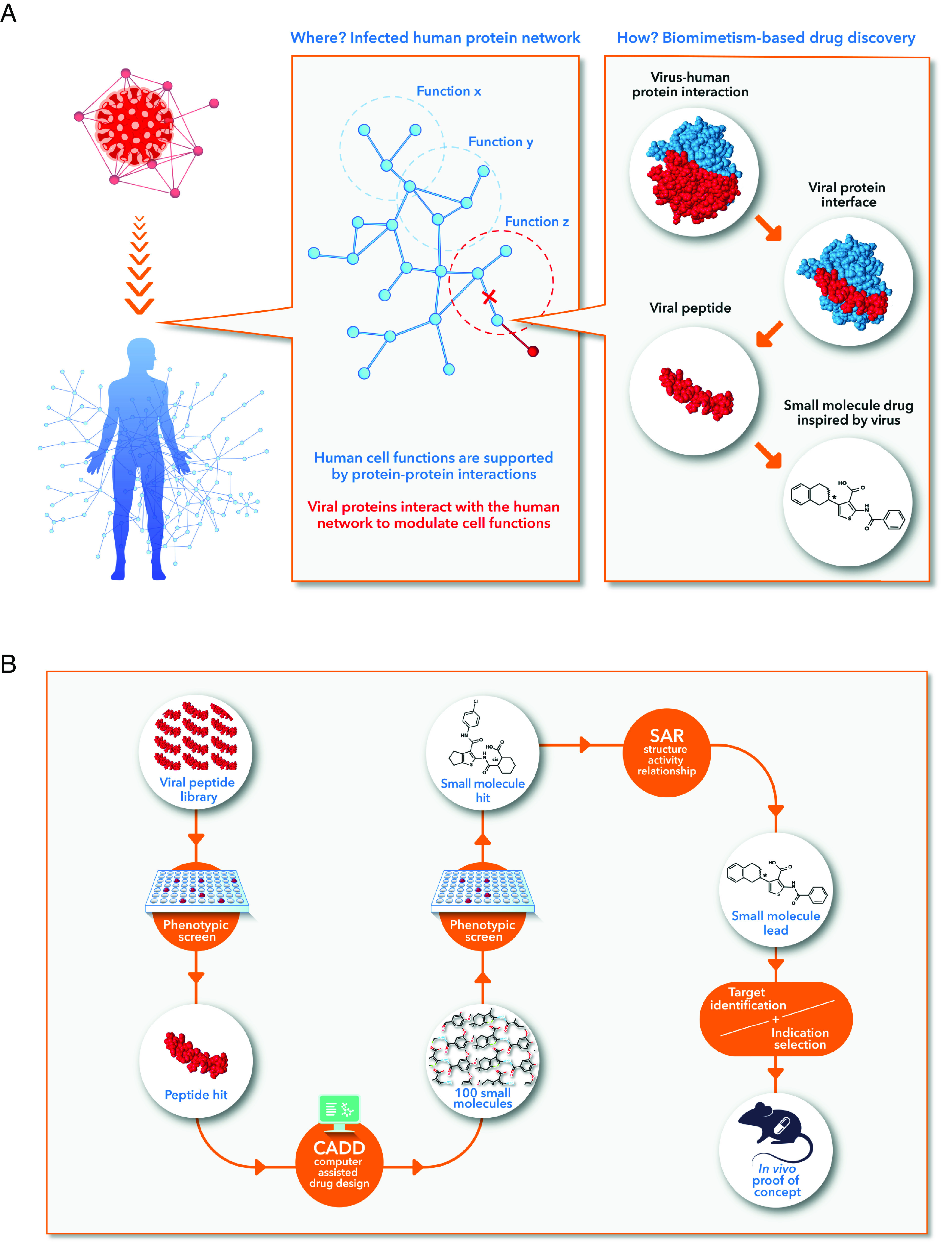
Principle of the drug discovery process inspired by viruses. (*A*) Viruses and human as networks of interacting proteins (*Left*). Following infection, a viral protein modulates a cellular function by interacting with a human protein (*Middle*). Interactions can be mapped to the level of small interfaces of 5 to 20 amino acids (*Right*). These peptides can be used as new chemical entities or translated into small molecules. Overall, viruses highlight manipulable human proteins and provide starting points to modulate their functions. (*B*) Sequential steps of the drug discovery process. The viral peptide library is screened in a cell-based assay. The pharmacophores of a peptide hit are translated into a library of small molecules by computer-assisted drug design. After the screening of the library, each hit becomes a matrix for a structure–activity relationship (SAR) process for the selection of a lead. The target identification is then a prerequisite to select the therapeutic indication.

This work describes a biomimetic process inspired by viruses to generate molecules, peptides, and small molecule drugs, targeting host proteins to modulate cellular functions (Fig. 1*A*).

## Results

### Overview of Our Drug Discovery Framework Inspired by Viruses.

Our framework of drug discovery integrates four consecutive steps (Fig. 1 *A* and *B*) starting with 1) the delineation of a comprehensive atlas of vh-PPI manually curated from the literature with annotation of interacting domains. 2) The extraction of small viral protein interfaces, typically peptides of 5 to 20 amino acids, optimized by evolution to interact with human proteins. These peptides form a library of new chemical entities that are screened in cell-based assays. 3) The translation of the pharmacophores of one of the most active peptides into a library of small molecules. Following the screening of this library, the best hit molecules integrate a SAR optimization process. 4) The lead compound is used to identify the cellular target of the series that allows the identification of the animal disease model where the molecule can be repositioned.

### The Viral Infection Landscape.

**Fig. 2.**
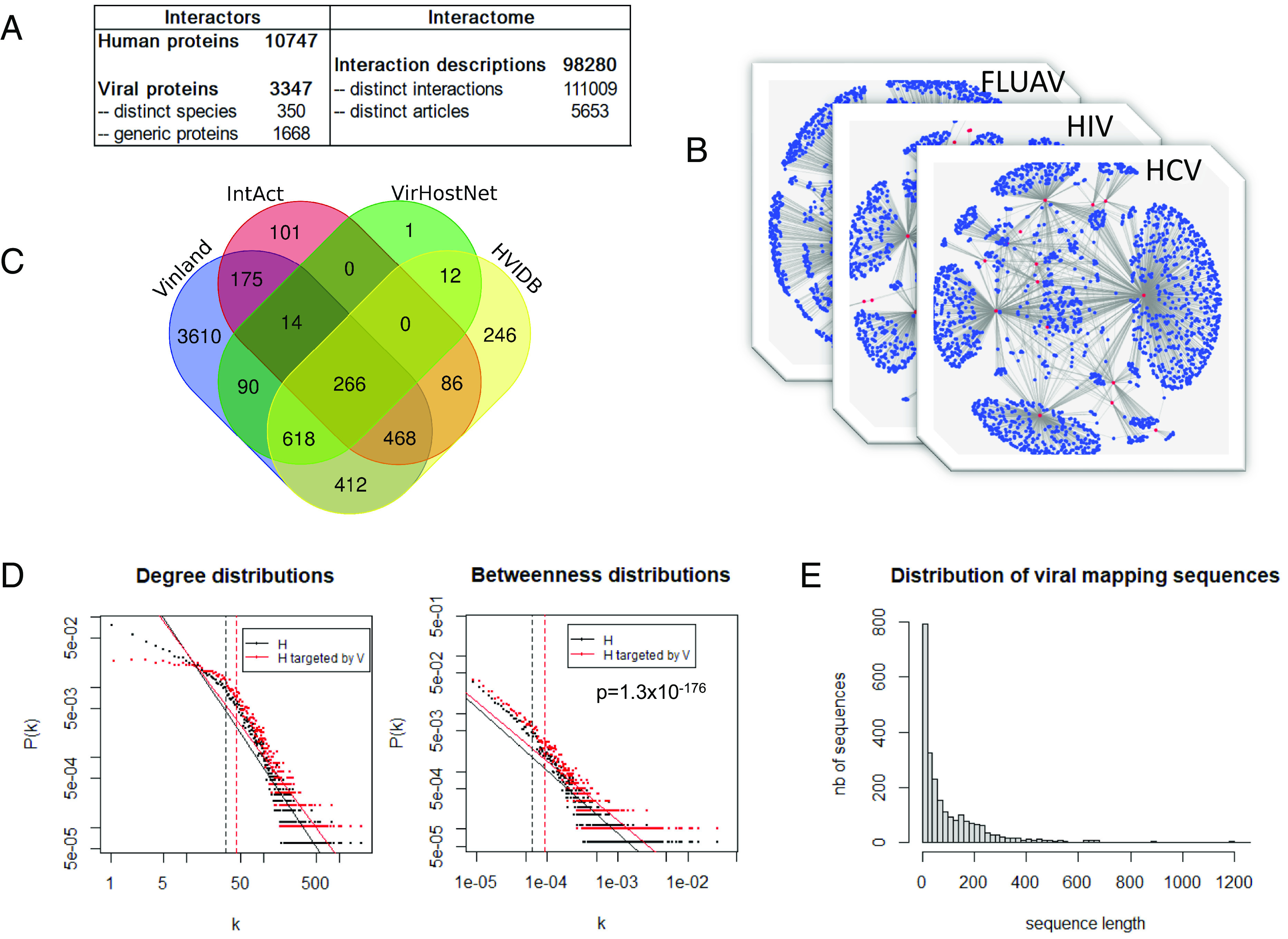
Description of the viral infection landscape in the Vinland database. (*A*) Key figures of the curated virus–human protein interactome integrated into Vinland. (*B*) Representation of interactomes between human and viruses of clinical importance. Blue dots: Human proteins. Red dots: Viral proteins. Gray edges: Physical protein interaction. (*C*) Venn diagram comparing the papers reporting at least one interaction between a human protein and a viral protein in Vinland, IntAct (12), VirHostNet (13), and HVIDB (14). (*D*) Topological analysis. *Left*: Degree distributions of human proteins (black) and human proteins targeted by viruses (red); P(k) is the probability of a node to connect to k other nodes in the network. Degree: Wilcoxon test, *P* = 3.2 × 10^−191^. *Right*: Betweenness distributions of human proteins (black) and human proteins targeted by viruses (red); P(b) is the probability for a node to have a betweenness value of b in the network. Betweenness: Wilcoxon test, *P* = 1.3 × 10^−176^. Solid lines are linear regression fits. Vertical dashed lines indicate the mean degree/betweenness values for each distribution. (*E*) Distribution of the length of interacting sequences on viral proteins.

The first pillar of our drug discovery framework is the viral infection landscape database (Vinland, freely accessible at https://www.vinland.network), an integration of all existing descriptions of vh-PPI, a knowledge accumulated by worldwide virology researchers. Years of manual stringent curation have indeed enabled the collection of all those interactions, which were finely annotated and then carefully integrated within Vinland (see curation process and database organization in *SI Appendix*). The interface allows one to easily navigate through the collection of interactions with the desired level of stringency, using a tabular format or an embedded network visualization tool. The database currently includes ca. 100,000 vh-PPI descriptions involving 10,700 human proteins and 3,300 viral proteins from 350 taxa (Fig. 2*A*). The landscape encompasses viruses from most groups of the Baltimore classification with a bias toward viruses of clinical importance (e.g., influenza virus, HIV1, HCV; Fig. 2*B*). Vinland represents a substantial upscaling of publicly available vh-PPI datasets (10, 11) (Fig. 2*C*) with more than 3,000 articles not identified elsewhere.

As previously observed on much smaller datasets (15, 16), viral proteins preferentially interact with hubs and bottlenecks in the human protein interaction network (Fig. 2*D*). This propensity to target the weaknesses of the human interactome is a powerful strategy to interfere with pathways and communication networks (17). In addition, proteins targeted by at least three different viruses are even more central in the human interactome suggesting similar coevolution strategies allowing viruses to target key biological processes.

Vinland also offers the opportunity to identify the cellular functions that are significantly targeted by viruses. Functional enrichment analyses were performed for the 50 viruses which are known to interact with more than 150 human proteins, using full Gene Ontology (GO) biological processes as well as a cut-down subset of GO (GOslim) biological processes, in order to provide a broader overview of the functional information without the details of the specific fine-grained terms (Dataset S1) (18, 19). At least one member of each Baltimore group is represented in this analysis. Over 4,200 GO and 200 GOslim biological processes are targeted by at least one virus, showing the large diversity of cellular functions that can be manipulated (*SI Appendix*).

To extend the interest of this vh-PPI dataset toward noninfectious diseases, we analyzed the set of cellular targets in regard to susceptibility genes. 63.2% of proteins coded by genes involved in human diseases, retrieved from DisGeNET (20), are also targets of viral proteins, showing a high statistical enrichment compared to proteins not targeted by viruses (exact Fisher test, *P* < 2.2 × 10^−16^). This relation can provide molecular basis to the pathophysiology associated to viral infections (21, 22). In a drug discovery perspective, this also indicates that the vh-PPI dataset could be used to mimic viral strategies for manipulating essential cellular functions that are impaired in diseases. Analyzing human protein sequences interacting with viral proteins reveals that these sequences are highly enriched in amino acids whose mutations are associated with diseases according to DisGeNET (exact Fisher test, *P* < 2.2 × 10^−16^). This highlights the potential of viral proteins to precisely target human proteins at sites that are pivotal for their activity.

Overall, the Vinland database has collected unique information on how viruses, through evolution, have developed tools to modulate the activity of cellular proteins involved in numerous biological processes.

### Viral Peptide Interfaces as New Chemical Entities to Target Cellular Functions.

In addition to identifying manipulable cellular targets, the Vinland database also integrates the mapping of interactions. The distribution of the length of interacting viral sequences (Fig. 2*E*) ranges from 5 to 1,200 residues (on average 15% of the protein). Viral peptides of 5 to 20 amino acids represent 728 sequences which are likely close to the minimal domain required to interact with human proteins. As expected, some of these viral peptides are also eukaryotic types of Short Linear Motifs (SLiMs) known to mediate modular interactions with human proteins (5, 6). Interestingly, viral peptide sequences are found significantly more covered by SLiMs, as described in the Eukaryotic Linear Motif resource (23), than viral protein sequences outside of the peptides (Fisher exact test, value <2.2 × 10^−16^). Furthermore, 232 of the 728 Vinland viral peptides (31.9%) contain a SLiM (Dataset S2). Hence, many other peptides that do not correspond to a known motif are likely ex nihilo viral interfaces which modulate the function of human proteins.

Through their ability to target human proteins, the pool of virus-derived peptides can be considered as new chemical entities per se, regardless of the virus and even of the viral protein from which they have been identified (Fig. 1*B*). Thereby, each viral peptide can be used to directly modulate the cell protein function. In addition, and as stated above, given the viral proteins’ propensity to interact with disease-related host proteins, these virus-derived peptides are invaluable molecules to target not only infectious diseases but also nonviral diseases. Thus, a sample library of 154 virus-derived peptides, in fusion with a HIV1 Tat protein cell-penetrating sequence, was synthesized. These peptides were the first peptides identified during the curation process. They are found by the scientific community to be necessary and sufficient sequences to target 149 human proteins (*SI Appendix*, Table S1) involved in diverse biological processes, related to diseases such as cancer, infectious diseases, and immunological diseases (*SI Appendix*, Table S2).

Interestingly, the pharmaceutical industry has developed experimental to FDA-approved drugs for only 42 of these proteins (24), so that the majority (71.8%) of the peptide targets are untapped. The conventional way to validate proteins as target candidates is to study the cellular impact of the knock-out or the knock-down of the expression of their genes. This dramatically perturbs the local protein interaction network. Conversely, virus-derived peptides, like small molecules, are expected to have a reduced topological impact. From this network perspective, peptides should theoretically disrupt the interaction between their target and another protein in the human interactome (25). They can also interfere with the catalytic activity of a human enzyme (26).

**Fig. 3.**
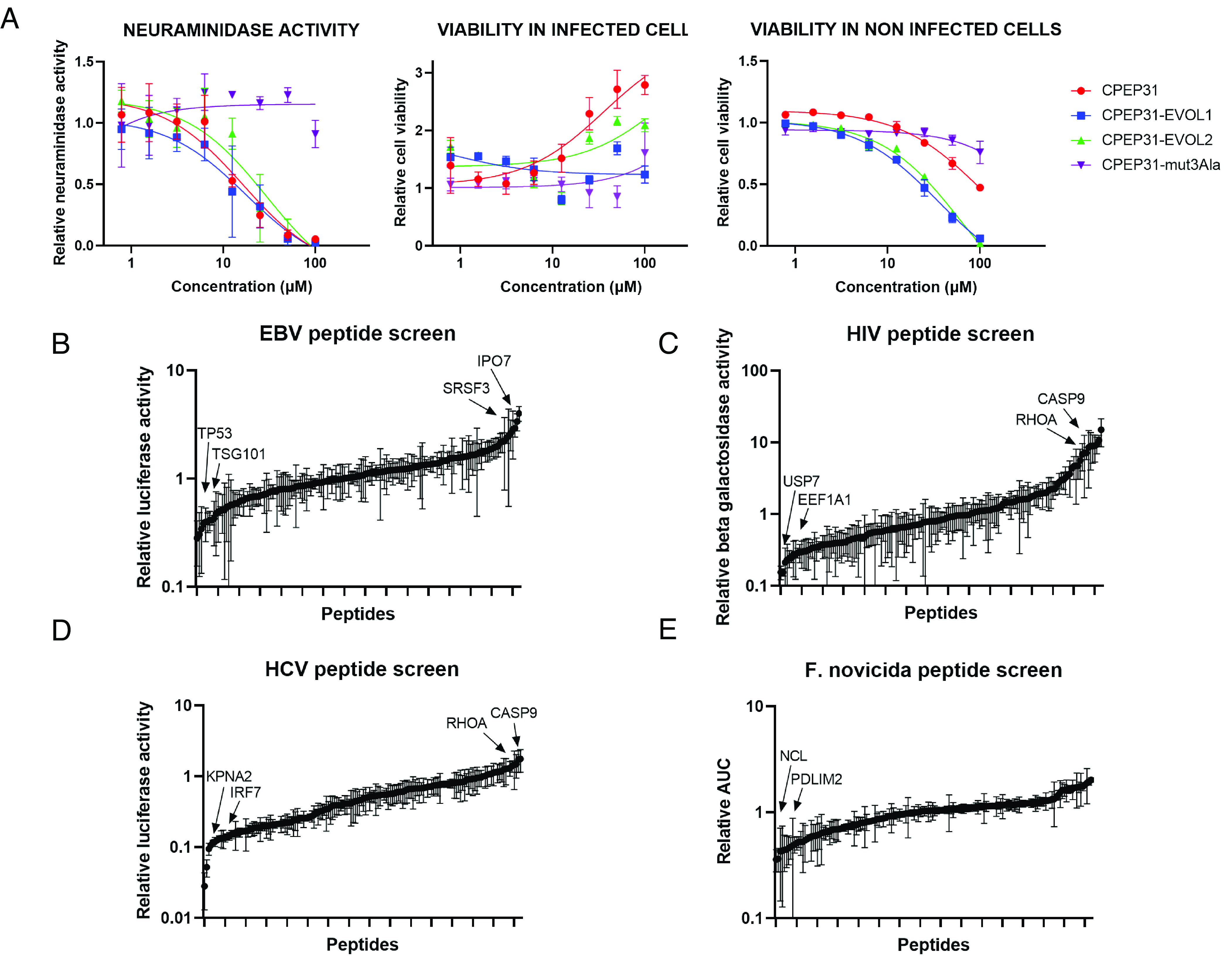
Viral peptide library screens. (*A*) Anti-influenza virus activity of CPEP31 peptide. Neuraminidase (NA) activity measured in the supernatant of A549 cells infected with influenza A H1N1 virus and treated with CPEP31 and derivative peptides. Cell viability is measured by the resazurin assay in infected and noninfected cells. Values are normalized to those of cells treated by vehicle (N = 3). CPEP31-mut3Ala is CPEP31 where LFL amino acids are mutated in AAA. CPEP31-EVOL1 and CPEP31-EVOL2 are CPEP31-similar peptides in vFLIP proteins from HVS and BoHV-4 viruses (YCLLFLINGC and FVMYFLLDPY respectively, in fusion with the cell-penetrating sequence). (*B*) Relative peptide activities in EBV screen. Late gene reporter transcriptional activation is quantified 48 h post-TPA and BA treatment in Hone-1 cells treated with 20 µM of peptide. (*C*) Relative peptide activities in HIV1 screen. Replication is monitored by measuring β-galactosidase activity in HeLa P4 cells carrying a Tat-inducible LacZ gene and treated with 20 µM of peptide. (*D*) Relative peptide activities in HCV screen. The HCV replicon RNA copy number in cells is evaluated by measuring the firefly luciferase activity in Huh7 and treated with 20 µM peptide. (*E*) Relative peptide activities in *Francisella novicida* screen. The AUC is calculated from PI incorporation signal in function of time in *F. novicida* infected BMDMs treated with 20 µM of peptide. (*B*–*E*) Data are normalized to signal with vehicle only. Arrows indicate peptides for which targets are commented on in *SI Appendix*.

Through its ability to modulate cell functions, we anticipated that the peptide library could be used to identify modulators of pathogen replication. The library was first tested in a dose–response experiment against influenza A virus replication in A549 cells. Almost 20% of peptides (n = 29) induced at least a twofold reduction of influenza A virus replication at the highest concentration (the dose–response curves for all peptides are shown in *SI Appendix*, Table S3). Strikingly, these peptides were identified on viral proteins unrelated to influenza virus. This illustrates that distant viruses can modulate cellular functions required for the replication of influenza A virus (*SI Appendix*, Table S1). As flagship of our approach, CPEP31 was the most active peptide with an EC50 concentration of 8.3 μM and a CC50 concentration above 100 μM which translates into a selective index (SI = CC50/EC50) superior to 12 (Fig. 3*A*). It was synthesized from a sequence of KSHV vFLIP protein corresponding to the first death effector domain and is reported to target ATG3 and CFLAR (27). Interestingly, ATG3 knock-out is independently reported to block influenza virus replication (28).

CPEP31 3 hydrophobic core residues (LFL) were mutated in three alanines to synthesize CPEP31-mut. These substitutions abrogate the binding of the peptide to its target (27). Accordingly, CPEP31-mut was no longer able to inhibit influenza replication (Fig. 3*A*). Homologs of KSHV vFLIP exist in other gammaherpesviruses, and as a result, in vivo evolution of viruses may have naturally selected sequences that could show better potency than CPEP31 on influenza replication. Homologous KSHV CPEP31 peptides were synthesized using the vFLIP sequence of HVS and BoHV-4 (Fig. 3*A*). Both peptides were found as potent as CPEP31 in the replication assay but were less efficient in inhibiting virus-induced cell lysis and more toxic than CPEP31. So far, CPEP31 appears to be the best naturally evolved sequence of vFLIP for the inhibition of influenza virus replication.

We further demonstrated the large potential of the virus-derived peptide approach with results obtained after screening the peptide library against other important groups of the Baltimore classification of viruses (HCV, HIV1, and EBV; Fig. 3 *B–D*). Peptide activities in different screens are listed in Dataset S3. In addition, to broaden the applicability of the virus-derived peptide library, it was screened outside the scope of a viral infection on the replication of the intracellular bacterium *F. novicida* in primary macrophages (Fig. 3*E*). These screens led to the identification of many functionally relevant peptide target associations that would deserve more explorations for anti-pathogen indications (*SI Appendix*).

This analysis reveals that virus-derived peptides are unique molecular entities designed by evolution, and able to modulate biological processes. They open the way to address proteins coded by genes involved in human diseases and cellular proteins hitherto considered as undruggable for infectious as well as noninfectious disease indications.

### Viral Peptide Translation into Small Organic Molecules.

**Fig. 4.**
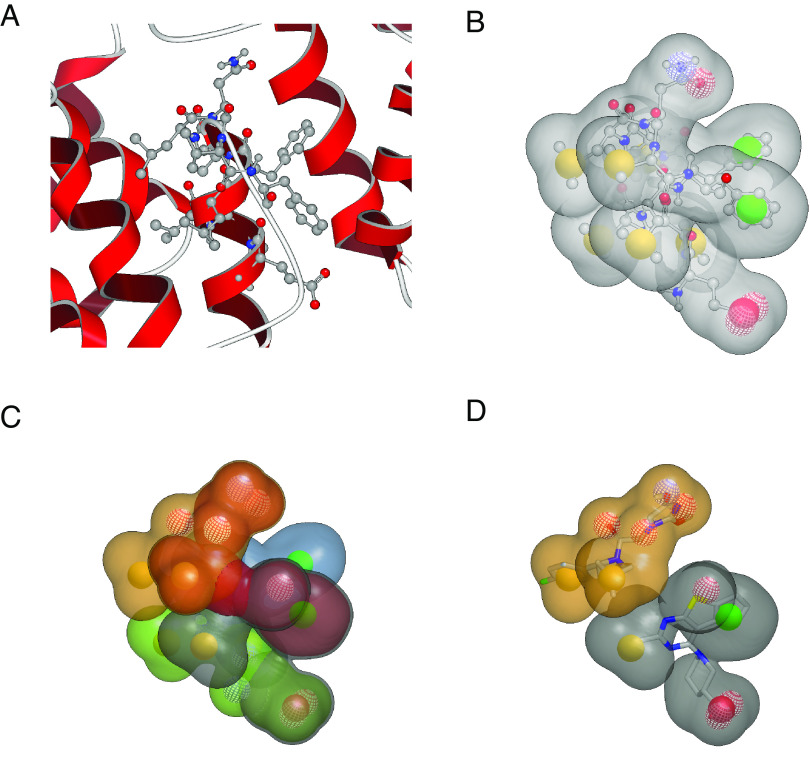
Ligand-based drug design of a library of small molecules from a bioactive peptide. (*A*) 3D structure of the peptide CPEP31 (displayed as balls and sticks) as a part of the protein vFLIP, (PDB entry 3CL3) represented as cartoon ribbon. (*B*) Energy-minimized conformation of peptide CPEP31, its shape is displayed in gray and major pharmacophoric points as colored spheres (yellow: hydrophobic, green: ring, solid red: negative ionizable, mesh red: H-bond acceptor, and mesh blue: H-Bond donor). (*C*) Overlay of five different rocs (32) queries with their shape highlighted in color (yellow, red, blue, green, and gray) and pharmacophoric points as colored spheres (same color code as in *B*). Each individual query covers only a part of the external surface of the peptide. Taken all together, they reproduce the overall shape of the full peptide. Six other queries are not represented in this picture for the sake of clarity. (*D*) Overlay of two rocs queries (shapes in gray and yellow) with 3D structures of an identified molecular hit shown as gray sticks.

A virtual screening approach which aims at finding small organic molecules able to mimic the CPEP31 peptide should rely solely on its own structural information and may ignore prior knowledge of potential protein targets. CPEP31 is a peptide of ten amino acids (sequence = EVVLFLLNVF), extracted from the KSHV vFLIP protein. vFLIP 3D structure was determined by X-ray [Protein Data Bank (PDB) entry 3CL3 (29)]. The first six residues form an α-helix and the last four remain folded as a loop (Fig. 4*A*). First, a simple geometry optimization of the peptide was performed to eliminate the small intramolecular interactions occurring between the peptide and the rest of the structure of the protein, as well as to position the side chains in an energy-minimized conformation. To convert this optimized structure into small organic molecules, a screening by shape was implemented (30). The full peptide shape was divided into a set of smaller overlapping ones. This approach which consists in dividing the large shape of a peptide into smaller ones works under the assumption that only a small region of the peptide is mandatory for its functional activity. In principle, it could be applied for larger peptides as long as their 3D structure is well-ordered and stable. Thus, eleven queries for the software rocs (31, 32) were built, each composed of shape and “colors” (pharmacophoric features such as hydrogen-bond donors, acceptors, and hydrophobic groups, as defined in Fig. 4). Each query was based on the side chains of three residues which are close in space and account for the external surface of the peptide (examples are displayed in Fig. 4*C*). With such a size, each individual shape query is well-suited to be fitted by molecules of molecular weight in the 300 to 500 Da range. Taken together, all the queries cover the complete solvent-accessible surface of the peptide and may be seen as surrogates of the full peptide shape, as seen when comparing Fig. 4 *B* and *C*. Each query was used to search a multiconformer database of about 6 million commercially available molecules with rocs (31, 32) yielding molecules in a 3D conformation which share the same shape and pharmacophoric features as the query. The top 0.5% scoring molecules (examples in Fig. 4*D*) were further filtered to ensure that the alignment with the query complied with additional requirements (*SI Appendix*). The resulting list contains about 400 molecules. Finally, they were all visually reviewed when superimposed on the structure of the peptide in 3D and in 2D by a medicinal chemist to ensure there was no undesirable or chemically reactive moiety. In this final selection, each individual rocs query is represented by a few molecules, ensuring that the coverage of the peptide’s full shape is complete. In the end, about 100 molecules passed all the steps and were purchased to be tested in the influenza virus replication assay (*SI Appendix*, Fig. S1).

### Hit Identification and Optimization.

**Fig. 5.**
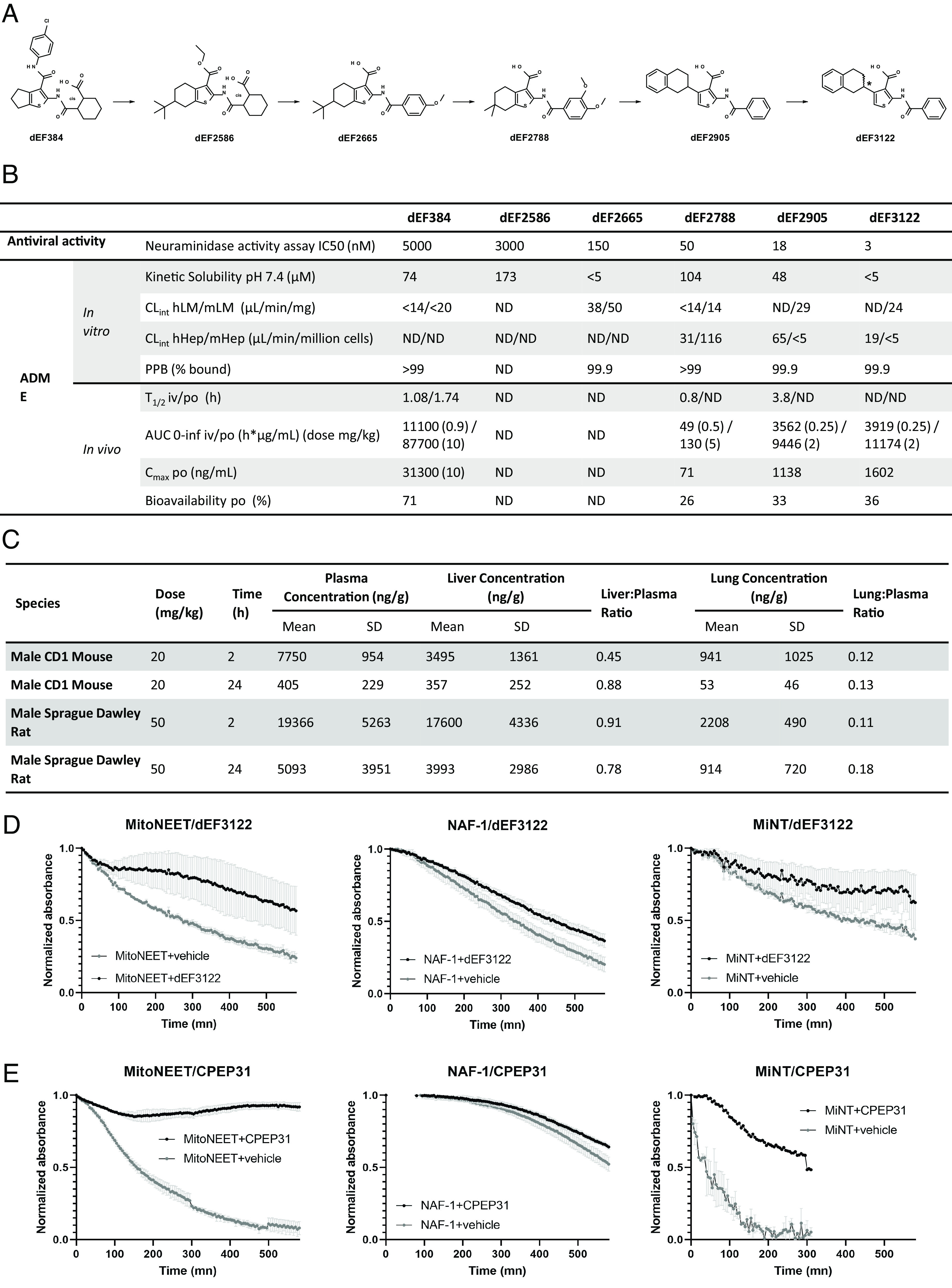
SAR, ADME, and interaction with NEET proteins during optimization. (*A*) Chemical structures of molecules inspired by CPEP31 from dEF384 to dEF3122 including main intermediates with key chemical modifications. (*B*) Antiviral activity and ADME (absorption, distribution, metabolism, and excretion) data of main compounds: IC50 from the neuraminidase activity assay in the supernatant of A549 cells infected by H1N1 influenza A virus (nM), kinetic solubility at pH 7.4 in µM, CL_int_ (intrinsic clearance) in hLM/mLM (human/murine liver microsomes) in µL/min/mg protein, CL_int_ (intrinsic clearance) in hHep/mHep (human/murine hepatocytes) in µL/min/million cells, plasma protein binding (PPB) (% bound) murine, mouse T_1/2_ (h-hours, iv-intravenous, po-per os), AUC 0-∞ (area under the curve) in h*µg/mL, C_max_ (maximum concentration) po in ng/mL and po bioavailability in %, (ND-not determined). (*C*) Tissue distribution of dEF3122 in mouse and rat. (*D* and *E*) [2Fe–2S] cluster release from the three recombinant NEET proteins at 37 °C was determined by following the absorbance of the cluster at 460 nm as a function of time for dEF3122 (*D*) and CPEP31 (*E*).

Among the library of molecules mimicking the CPEP31 peptide pharmacophores, three compounds bearing the 2-amidothiophene-3-carboxamide substructure emerged as a major hit class, including dEF384 (Fig. 5 *A* and *B*) with an IC50 of 5 µM for the inhibition of influenza virus replication. Optimization of this hit through medicinal chemistry was then undertaken (*SI Appendix*). In order to gain more SAR insight around this compound, a second round of commercial compound acquisition based on structural analoging of dEF384 enabled the identification of the compound dEF2586 with improved ligand efficiency (similar potency for a reduced size).

The shift from 3-benzamide to 3-ethyl ester dEF2586 (IC50 of 3 µM) showed that the bulk of the arylamide in that area was not productive. Classical medicinal chemistry program through iterative design, synthesis, and compound testing for their inhibition of viral replication was then performed and allowed to progress compound properties. The main advances achieved during this project are highlighted with the dEF2665, dEF2788, dEF2905, and dEF3122 compounds which display significant breakthroughs among their synthesized analogs. Hence, the release of the carboxylic acid from the ester group was tolerated, provided that the modification of the 2-amide moiety was performed simultaneously to exchange the second carboxylic group with a less polar function in order to retain cell permeability. This led to the identification of dEF2665 and its benzamide analogs. Such a twist modification enabled a breakthrough in potency (dEF2665 IC50 down 20-fold to 0.15 µM).

Optimizing the tetrahydrophenyl left part of the molecule by replacing the exocyclic tert-butyl moiety with a gem-dimethyl group provided a further increase in potency with compound dEF2788 (IC50 of 50 nM) and removed the unnecessary chiral center. Nevertheless, this advanced compound remained too lipophilic and suffered from metabolic stability liabilities as well as low mouse plasma exposure (Fig. 5*B*), making it unsuitable for in vivo testing. dEF2788 showed sufficient potency to search for the target, an essential step indicated in Fig. 1*B*. This is described in a previous article using a photoaffinity labeling-based method where we identified the NEET family of protein: MitoNEET, NAF-1, and MiNT (33).

The improvement of the plasma exposure was achieved by replacing the tetrahydrophenyl fused left part of the molecule with the unfused tetralin moiety as in dEF2905, still maintaining a very high potency (IC50 of 18 nM). The interaction of dEF2905 with the NEET proteins was also validated in a recent publication from our team by different methods, including X-ray crystallographic analyses (34). Both stereoisomers were shown to interact with these proteins.

Chiral HPLC separation of stereoisomers followed by crystallization and X-ray diffraction indicated a more active enantiomer (dEF3122). For this molecule, both antiviral potency (IC50 of 3 nM) and PK properties were improved, with low clearance and high exposure in mouse plasma. The oral bioavailability for dEF3122 was 36% after oral administration at 2 mg/kg, with a plasma concentration reaching 1,602 ng/mL, equivalent to 2.6 µM (Fig. 5*B*). The tissue distribution was also assessed at two different time points (2 and 24 h) after per os administration in mice (20 mg/kg) and rats (50 mg/kg) (Fig. 5*C*). These results demonstrated that dEF3122 underwent a rapid and wide distribution to the liver and plasma compared to the lung. Such low lung/plasma ratio precluded the use of dEF3122 in respiratory diseases such as influenza without further optimization. However, this compound gradually and equally decreased to a very low concentration in all three tissues, reflecting the absence of accumulation. The rapid and extensive distribution of the lead compound dEF3122 to the liver suggests that it could be of great interest in therapies targeting this organ.

The series optimization based on a phenotypic screen is associated with a risk that optimized analogues may act through a different target (35). It is therefore mandatory to identify the target during the SAR process (Fig. 1*B*). As stated above, we identified the NEET family of human [2Fe-2S] proteins (MitoNEET, NAF-1, and MiNT) as the target of dEF2788 (33) and we confirmed that this target is maintained for dEF2905 (34). In these studies, interaction with NEET proteins is systematically validated by a [2Fe-2S] cluster release assay. Thus, we also tested and validated the interaction between dEF3122 and the NEET proteins using this assay (Fig. 5*D*).

At first sight, the targets may look surprising as the original virus-derived peptide used as a matrix for the drug design was described to bind CFLAR and ATG3 (27). As the virus–host interactome is far from being complete, we retrospectively tested CPEP31 interactions with the NEET proteins. Interactions were validated again using the cluster release assay for all three NEET proteins (Fig. 5*E*). Thus, we demonstrated that the NEET family of human proteins remains a target of the series of molecules described in this work, from the original matrix CPEP31 to the most optimized small molecule dEF3122.

### Therapeutic Application in an In Vivo Mouse Model.

One great value of our drug discovery process (Fig. 1*B*) lies in the viruses’ preferential targeting of disease-related human proteins. So, following the identification of the target, one major indication for the molecule is a disease related to this target. Here, NAF-1 is a recently identified mitochondrial drug target for nonalcoholic steatohepatitis (NASH) (36, 37). This disease is associated with liver damage and inflammation, and its severity is also associated with an increased risk of chronic kidney disease (38). To this day, there are still no approved therapeutics to treat NASH and associated kidney damages. As dEF3122 targets NAF-1 and is largely distributed in the liver, it appears as an ideal candidate to be tested in a diet-induced obesity model of NASH (39).

**Fig. 6.**
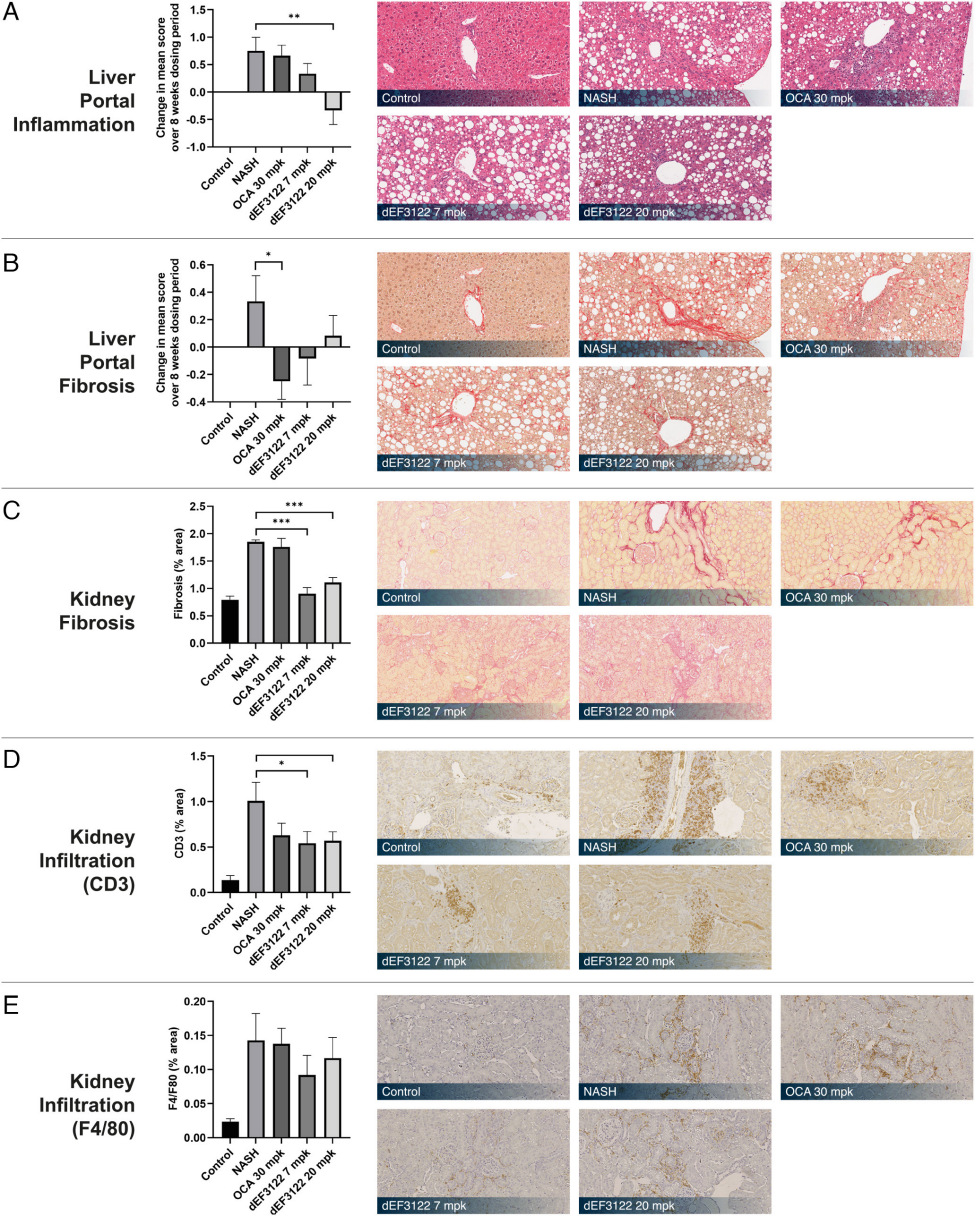
Effect of the lead compound in a diet-induced obese model of NASH. Mice fed with a high fat, high fructose, and cholesterol supplemented diet for 38 wk were then treated in the last 8 wk with vehicle, dEF3122, or OCA (obeticholic acid) at indicated concentrations (n = 12 animals per group). (*A* and *B*) Histopathological mean score changes between the beginning and end of the treatment for liver portal inflammation (*A*) and liver portal fibrosis (*B*) are shown with representative stained liver sections of hematoxylin and eosin (H&E) and picrosirius red stains, respectively. (*C*–*E*) (*C*) Areas of kidney fibrosis at the end of the treatment are shown as % of the total area of the selected fields with representative sections of the kidney stained with picrosirius red. (*D*) % CD3 and (*E*) % F4/80 positive areas in kidneys with representative sections of CD3 and F4/80 immunostainings. *P < 0.05, **P < 0.01, and ***P < 0.001 from the *T* test with a preliminary nonsignificant Shapiro–Wilk test or from Wilcoxon signed–rank test otherwise.

Here, mice fed amylin liver non-alcoholic steatohepatitis, NASH (AMLN) diet (40) for 38 wk and treated by vehicle in the last 8 wk presented histological features of NASH, which includes significant liver inflammation and fibrosis in the portal region (Fig. 6 *A* and *B*). Portal inflammation is a key feature of disease progression and is associated with fibrosis stage (41). In addition, kidney sections exhibited severe renal lesions such as interstitial fibrosis, macrophages, and lymphocytes infiltration (Fig. 6 *C–E*). Overall, this mouse model recapitulated phenotypes of NASH associated with chronic kidney disease.

Compared to the vehicle group, oral administration of dEF3122 for 8 wk resolved portal inflammation and fibrosis in the liver (Fig. 6 *A* and *B*). Strikingly, while the positive control OCA (39) also significantly reduced portal fibrosis, it failed to alleviate portal inflammation. In the kidney, dEF3122 dramatically reduced fibrosis and attenuated interstitial lymphocytes infiltration but not macrophages infiltration (Fig. 6 *C–E*).

Altogether, the positive therapeutic results of dEF3122 in the NASH mouse model fully validate this drug discovery approach, which starts from the knowledge of virus–host protein interactions and goes all the way to the identification of original small molecule drugs to modulate untapped targets and cellular functions.

## Discussion

Inspiring our drug discovery approach, viruses identify manipulable cellular targets and provide structural information on how to manipulate them. They should be regarded as a small yet highly effective version of drug developers that nature has been optimizing through millions of years of evolution. The natural virus–host coevolution has selected highly rare viral interfaces that would be hard to identify using conventional methods such as peptide arrays or phage display. The use of viral peptides as new chemical entities to modulate cellular functions has proven its efficiency in this work after screening of a peptide sample library, with the identification of numerous modulators of the replication of various pathogens. In addition, most virus-derived peptides interact with cellular proteins that are not yet tapped by molecules, opening large perspectives in term of drug discovery.

As described in Fig. 1*B*, the target identification occurs when the small molecule series reaches a high level of potency. This is a mandatory step when a phenotypic screen is used as a primary assay for the SAR process (35). Here, while we show that the NEET proteins remain the targets from the peptide to the most optimized molecule, these targets were not anticipated. Neither CPEP31 nor VFLIP have been described to interact with these proteins (27), see in Vinland. This is an example of the incompleteness of the virus–host protein interactome. Consequently, a peptide should be considered as an evolutionary-designed surface of interaction with human proteins and a matrix for drug discovery. The chemoinformatic approach to design the library of 100 small molecules is based on the structure of the peptide and is not inspired by the structure of the target. The peptide target should only be treated as one of the potential targets for the small molecule series.

Here, as a proof of concept, our drug discovery approach identified the lead molecule dEF3122, designed by virtual screening and subsequent medicinal chemistry optimization, that showed an interesting activity in the NASH mouse model in the liver and in the kidney. A recent study of a nonalcoholic fatty liver disease human cohort reports a relationship between portal inflammation and renal dysfunction (42). Patients with a higher score for liver portal inflammation had a 7.7-fold increased risk of renal issues. While improving portal inflammation, dEF3122 also decreased renal fibrosis and mononuclear cell infiltration in the kidney. This further validates the association between portal inflammation and renal injuries at the histological level and suggests that molecules resolving portal inflammation, such as dEF3122, are therapeutic candidates for NASH patients with renal complications.

For the use of peptides, their development toward preclinical models faces several challenges including their high susceptibility to proteolytic degradation, the delivery route, their potential immunogenicity, and the cost of manufacturing. This is well-reflected by the low number of clinical trials testing cell-penetrating peptides. While many of these hurdles are being tackled with promising results (43), the development of such therapeutics is still in its infancy. In this perspective, the best active peptides in Fig. 3*D* should be stabilized and optimized or should preferentially follow the full drug discovery process described here before a test in a mouse model. Nevertheless, these virus-derived peptides are of great interest to target cellular proteins, including those untapped by the pharmaceutical industry (24). They can be used in addition to molecular approaches modulating gene expression to explore a cellular function in any cellular context.

Regarding the small molecule drugs described here, given their liver distribution their antiviral potential against hepatotropic viruses is very attractive. We showed previously that chemical targeting of NEET proteins with dEF2788 (one intermediate of the SAR described in Fig. 5 *A*–*C*) modifies mitochondrial morphodynamics by stabilizing ER-Mitochondria contact sites and can counteract virus-induced mitochondria network hyperfusion (33, 44). We further connected dEF2788 antiviral activity to the innate immunity signaling RIG-I complex at mitochondria. This molecule shows not only anti-influenza virus activity but is also active in vitro against Dengue virus replication. As described here, for PK reasons dEF2788 is not suitable for in vivo experiments, contrary to dEF3122 (Fig. 5 *B* and *C*). Therefore, in vitro and in vivo exploration of dEF3122 for anti-hepatotropic virus activity is really appealing and will be tested in the future.

Overall, taking advantage of decades of collective work of the scientific community hunting for virus–host protein interactions, besides providing invaluable information for virologists, Vinland is also a source of inspiration for drug discovery. By mimicking viral infection strategies, this study now provides molecular tools, peptides, and translated small molecules, which can be precursors of original drugs including for orphan targets. This approach is now starting to be used in drug discovery in multiple therapeutic areas beyond infectious diseases.

## Materials and Methods

### Network Representation and Analysis.

Networks are visualized with Cytoscape (45) using a force-directed layout. The R statistical environment was used to perform statistical analysis (46). Network topology measures were computed with the igraph R package (47).

### Gene–disease and Variant–disease Associations.

Curated gene–disease associations were downloaded from DisGeNET (20) and filtered with a minimum confidence score of 0.4. The resulting gene list was compared with genes coding for proteins interacting with viral proteins.

Curated variant–disease associations were also downloaded from DisGeNET. Single nucleotide polymorphisms (SNPs) were compared to the SNPs known in the human proteins of Vinland according to the UniProt reference (48). SNPs associated with diseases were distributed in two groups defined either by their occurrence in a domain mapped in interaction with viral proteins or not.

### HCV Assay.

Huh7 human hepatoma cells were grown in Dulbecco’s modified Eagle’s medium (DMEM) medium supplemented with 2 mM L-glutamine, nonessential amino acids, 100 U of penicillin, 100 μg of streptomycin (Gibco), and 10% fetal calf serum. In the R1b subgenomic replicon, the region encoding the structural proteins of HCV is substituted by the firefly luciferase gene downstream of the HCV IRES. This region is followed by the encephalomyocarditis IRES upstream of the sequence encoding the NS3-NS5B polyprotein (49). HCV replicon transcripts were synthesized as described previously (50) and electroporated in cells. Huh7-transfected cells were seeded into 96-well plates at a density of 10^3^ per well and treated with 20 µM of peptide. Three days posttreatment, luciferase assay was carried out using Luciferase Cell Culture Lysis and Luciferase Assay Reagents (Promega) and measured on a Top Count NXT counter (Perkin Elmer). Values are averages from triplicate cultures and are expressed as relative ratios of the values obtained 4 h after electroporation.

### *Francisella-*mediated Cell Death Assay.

Preparation and culture of bone marrow–derived macrophages (BMDMs) were performed as previously described (51). BMDMs were differentiated in DMEM medium (Invitrogen) with 10% v/v fetal calf serum (Thermo Fisher Scientific), 10% MCSF (L929 cell supernatant), 10 mM HEPES (Invitrogen), and 5% sodium pyruvate. One day before infection, macrophages were seeded into 96-well plates at a density of 5 × 10^4^ per well. For infections with *F. novicida* (strain U112), bacteria were grown overnight in TSB supplemented with 0.1% (w/v) cysteine at 37 °C with aeration. The bacteria were added to the macrophages at a multiplicity of infection (MOI) of 1. The plates were centrifuged for 15 min at 1,500 *g* and placed at 37 °C for 60 min. Cells were washed and fresh medium containing 10 μg mL^−1^ gentamycin (Invitrogen) was added with 20 µM of peptide. Cell death kinetics were monitored in BMDMs by assessing incorporation of propidium iodide (used at 5 μg/mL) in real time through measurement of fluorescence emission at 635 nm every 15 min on a microplate reader (Tecan Spark 20M) during 20 h. Kinetics were quantified by calculating the corresponding area under the curve (AUC) and subtracting the AUC of noninfected cells.

### Influenza Replication Assay.

Influenza replication assay was performed as previously described (43). Human lung adenocarcinoma (A549, ATCC) and Madin-Darby Canine Kidney (MDCK, ATCC) cell lines were grown in DMEM supplemented with 10% heat-inactivated fetal bovine serum (FBS, Pan Biotech) and 50 IU/mL penicillin G, 50 mg/mL streptomycin (Gibco), at 37 °C under 5% CO_2_. Influenza A virus A/New Caledonia/2006 (H1N1) (clinical isolate) was propagated in MDCK cells. A549 cells were seeded into 96-well plates at a density of 3 × 10^4^ per well and infected with influenza A H1N1 virus at a MOI of 1 in infection medium [DMEM serum-free medium supplemented with 50 IU/mL penicillin, 50 mg/mL streptomycin, and 0.2 mg/mL TPCK-trypsin (Sigma, ref 4352157)] with peptides or molecules at indicated concentrations. After 48 h, supernatants were harvested to test neuraminidase activity. This viral enzyme is able to cleave the 2′-(4-Methylumbelliferyl)-α-D-N-acetylneuraminic acid sodium salt hydrate (Munana, Sigma, ref M8639), generating a fluorescent product which can thus be quantified. In a 96-black plate, 25 μL of infection supernatants was diluted in 25 μL D-PBS containing Ca2+ and Mg2+, and the reaction started with 50 μL of 20 mM Munana. After 1 h incubation at 37 °C, the reaction was stopped by adding 100 μL of glycine 0.1 M and ethanol 25%. Fluorescence was recorded using a microplate reader (TECAN Spark 20M) at wavelengths of 365 nm for excitation and 450 nm for emission.

### HIV1 Replication Assay.

HIV-1 X4-tropic particles were produced in HEK293T cells transfected by the pNL4-3 construct. After 48 h, medium was recovered, aliquoted, and stored at −80 °C. Titration was performed using HeLa-P4 cells, which contain the LacZ gene under control of the integrated HIV-1 LTR (52). Serial dilutions of thawed viral stocks were used to infect Hela-P4 reporter cells. To determine viral titers, LacZ- foci were revealed 72 h later and counted for each dilution. Titers measured using this procedure reached 2 to 5 × 10^5^ infectious units/mL. For the infection assay with peptide treatment, Hela P4 cells were seeded in 96-well plates at a density of 5 × 10^3^ cells per well one day before infection. The day after, cells were treated by 20 µM of peptides and infected 3 h later with HIV1 strain NL4-3 particles at a MOI of 0.1. After 48 h of infection, cells were lysed with 30 µL lysis buffer (Tris-HCl and 0.05% IGEPAL), and viral replication was determined by Gal-Screen^TM^ assay (ThermoFisher, Ref T1027) and recorded using a microplate reader (TECAN Spark 20M).

### EBV Replication Assay.

The luciferase reporter gene was cloned downstream of the wild-type BdRF1 promoter (late gene promoter) and inserted in pHEBo plasmid (53) to create pHEBo:pLate-Luc to monitor late gene expression. Nasopharyngeal carcinoma Hone-1 cells were cultured in RPMI-1640 with 10% FBS and maintained in a humidified, 5% CO_2_ incubator at 37 °C. pHEBo:pLate-Luc plasmid was stably transfected into Hone-1 cells under a selective medium containing Hygromycin B (100 µg/mL). Induction of the EBV productive cycle was obtained by treating cells with TPA (20 ng/mL) and BA (3 mM), in the presence of 20 µM of peptides. Forty-eight hours later, cells were collected by centrifugation and washed in PBS, and luciferase activity was revealed using the luciferase assay system (Promega, Ref E4550) and recorded using a microplate reader (TECAN Spark 20M).

### Virtual Screening for Peptide Mimics.

Structure 3CL3 was downloaded from the PDB and prepared with the protein preparation wizard (54) within Maestro (55). The ten residues of CPEP31 were isolated and the structure was minimized using Macromodel (56) using the OPLS2005 force field. The energy-minimized structure was then used as a template to build rocs queries using the program vrocs. Each query was based on three residues chosen to be close in space and to represent a side of the peptide’s global 3D structure. Only the side chains of these residues were used to define the shape. Some pharmacophoric features (named colors in rocs) were used to complement some queries and were manually selected based on their solvent accessibility. Thus, features on the backbone of the residues which were not directed toward the external side of the peptide were excluded. On the other hand, features located on the side chains were kept. A total of eleven queries were built in that manner covering all sides of the peptide structure. Out of these, only one query did not involve at least one polar side chain. The virtual screening process is summarized in *SI Appendix*, Fig. S1. A multiconformer database, the Aldrich Market Select collection (about six million compounds), was first prepared using a maximum of fifteen conformers per molecule, generated with omega (57, 58). For each query, three searches were conducted with rocs, each time using a different metric to rank the hit molecules. The three metrics used were TanimotoCombo, ColorTanimoto (focus on the pharmacophoric features only), and RefTverskyCombo (where molecules can be larger than the query). For each of them, the best 2,000 molecules were kept. Then, the hit molecules were then filtered using Pipeline Pilot (59) to make sure that all three side chains used to build the query were covered and that no polar atom (O or N) laid near the centrist of the hydrophobic side chains. It was observed that most of the hits obtained with the ColorTanimoto and TanimotoCombo did not pass these filters. It was expected since these metrics tend to rank higher even partial shape/color hits over hits with too many features. The RefTverskyCombo metric is a good alternative to circumvent that effect as it allows the hit molecules to have extra features without penalizing them as long as the required ones are present. Molecules with a structure which was too simple (measured by the size of their FCFP_4 fingerprint) or too flexible (measured by their number of rotatable bonds) were discarded as well. The criteria were as follows: FCFP_4_size had to be greater than thirty, and the number of rotatable bonds had to remain equal to or under six. Next, all molecules were grouped into families using the “cluster molecules” component with the FCFP_4 metric and no a priori knowledge of the final number of clusters. Then, for each query and metric, a maximum of thirty molecules were retained by first keeping three molecules at most from each cluster and keeping the top scorers in the metric, complexity, and number of rotatable bonds. The total number of hits at that stage was just below 400. These molecules were visually reviewed by displaying the 3D conformation aligned to the query and looking at the superposition with the CPEP31 peptide. Molecules that were removed included ones which had a clear mismatch of polarity or shape, as well as those for which the conformation was estimated of high energy (e.g., R group in axial of a cyclo-hexyl group). Next, the 2D structures of the remaining hits were checked by a medicinal chemist to exclude the presence of a reactive/undesirable chemical group. Finally, the availability of 5 mg of compounds was checked using the online tool provided by Aldrich Market Select. The final list contained 102 molecules.

### Compound syntheses.

The compounds were made by Inventiva Pharma and Sygnature Discovery, companies experienced in Drug Discovery, according to the syntheses described in the patents (WO 2018/122317 for dEF384, WO 2019/154949 for dEF2586 and dEF2665, WO 2019/154950 for dEF2788, and WO 2019/154956 for dEF2905 and dEF3122), including chiral HPLC separation of stereoisomers from dEF2905 for the identification of dEF3122.

### In Vitro NEET Cluster Release.

MitoNEET (60) and NAF-1 (61) cytosolic domains and MiNT (62) were purified from *E. coli* as previously published. Experiments were performed in 96-well plates at 37 °C with 20 mM of Fe-S cluster in a buffer composed of 50 mM Tris-HCl pH 7 and 100 mM NaCl. dEF3122 was dissolved in dimethyl sulfoxide (DMSO) at 10 mM. CPEP31 was dissolved in PBS at 10 mM. After 20 min of incubation of the protein with the molecule, NEET cluster release was followed by measuring the absorbance at 460 and 800 nm over time using a plate reader (SpectraMax ID3). The absorbance at 460 nm was corrected for the variation of the baseline (absorbance at 800 nm) over time and normalized at 1 at the beginning of the kinetics.

### NASH Mouse Model.

Five-week-old male C57BL/6JRj mice were fed either chow diet or AMLN diet (40% total fat kcal of which 18.5% were trans-fat kcal, 20% fructose, and 2% cholesterol). This diet was maintained for 30 wk prior to study initiation. Three weeks prior to study initiation, liver biopsies were taken, and animals with steatosis grade <2 and fibrosis stage <1 were excluded from the study. Prior to the first administration of the test compound, stratified randomization of mice into treatment groups was performed according to collagen 1a1 (IHC) morphometry from the week 3 biopsies. Each treatment group consisted of 12 mice. For a total of 8 wk, compound dEF3122 was administered orally, twice daily at a concentration of 7 mg/kg or 20 mg/kg. OCA was administered orally, once daily at a concentration of 7 mg/kg. Animals were killed at the end of the treatment, and livers and kidneys were collected.

## Supplementary Material

Appendix 01 (PDF)

Dataset S01 (XLSX)

Dataset S02 (XLSX)

Dataset S03 (XLSX)

## Data Availability

All study data are included in the article and/or supporting information. Vinland database is freely accessible at https://www.vinland.network (63).
