# Supplementary material for: Viruses traverse the human proteome through peptide interfaces that can be biomimetically leveraged for drug discovery
Source: Proc Natl Acad Sci U S A. 2024 Jan 22;121(5):e2308776121. doi: 10.1073/pnas.2308776121 (PMC10835127; doi:10.1073/pnas.2308776121)
Supplement: Supplementary file 1 — Appendix 01 (PDF) [file pnas.2308776121.sapp.pdf]

Supporting Information for

Viruses traverse the human proteome through peptide interfaces that can be biomimetically leveraged for drug discovery

Laurène Meyniel-Schicklin[1], Jérôme Amaudrut[2], Pierre Mallinjoud[1], Fabrice Guillier[2], Philippe E. Mangeot[3], Laetitia Lines[1], Anne Aublin-Gex[3], Caroline Scholtes[3], Claire Punginelli[3], Stéphane Joly[1], Florence Vasseur[4], Evelyne Manet[3], Henri Gruffat[3], Thomas Henry[3], Farès Halitim[1], Jean-Laurent Paparin[1], Peter Machin[1†], Raphaël Darteil[1], Diane Sampson[1], Ivan Mikaelian[5], Lydie Lane[6], Vincent Navratil[7], Marie-Pierre Golinelli-Cohen[8], Fabiola Terzi[4], Patrice André[3], Vincent Lotteau[3], Jacky Vonderscher[1], Eric C. Meldrum[1], Benoit de Chassey[1]*

[1] ENYO Pharma SA, Lyon, Bioserra 1, Bâtiment B, 60 avenue Rockefeller, 69008, France.
[2] Inventiva, 50 rue de Dijon, 21121 Daix, France.
[3] CIRI, Centre International de Recherche en Infectiologie, Univ Lyon, Inserm, U1111, Université Claude Bernard Lyon 1, CNRS, UMR5308, ENS de Lyon, 21 Avenue Tony Garnier, Lyon, 69007, France.
4 Université de Paris, INSERM U1151, CNRS UMR 8253, Institut Necker Enfants Malades (INEM), Département "Croissance et Signalisation", 75015 Paris, France.
[5] Université de Lyon, Université Claude Bernard Lyon 1, INSERM 1052, CNRS 5286, Centre Léon Bérard, Centre de recherche en cancérologie de Lyon, 69373, Lyon, France.
[6] CALIPHO Group, SIB Swiss Institute of Bioinformatics, 1015 Lausanne, Switzerland.
[7] PRABI, Rhône-Alpes Bioinformatics Center, Université Lyon 1, 69622 Villeurbanne, France; European Virus Bioinformatics Center, 07743 Jena, Germany; Institut Français de Bioinformatique, IFB-core, UMS 3601, 91057 Évry, France.
[8] Université Paris-Saclay, CNRS, Institut de Chimie des Substances Naturelles, UPR 2301, 91198 Gif-sur-Yvette, France.
[†] Deceased.

*** Corresponding author:** Benoît de Chassey, **email:** benoit.dechassey@gmail.com

This PDF file includes:

- Supporting text
- Figure S1
- Tables S1 to S3
- Datasets S1 to S3 legends
- SI References

Supporting text

Curation process and database organization

The viral infection landscape dataset in Vinland results from 15 years of extensive and manual curation of the scientific literature to identify all existing descriptions of virus-human protein-protein interactions currently known. In addition, Vinland integrates a manually curated human-human protein-protein interaction (hh-PPI) dataset (see below) (also deposited in neXtProt at the Swiss Institute of Bioinformatics(1), essentially focusing on high-throughput screening studies and combined with the IntAct and neXtProt curated hh-PPI datasets. The global human protein interactome consists of 284,940 hh-PPIs between 17,235 human proteins and allows the exploration of the network topology of viral targets in the human interactome.

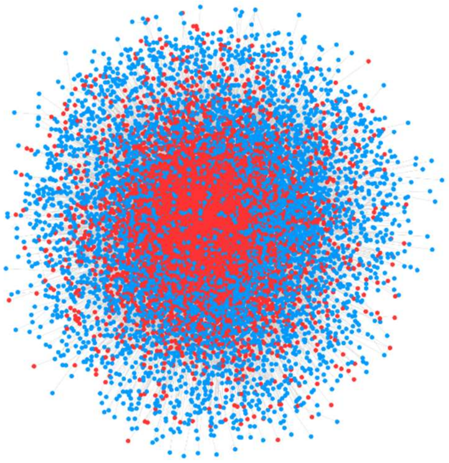

Representation of the human interactome. Dots: human proteins. Red dots: proteins targeted by a viral protein. Edges: physical protein interactions. The layout brings connected nodes closer.

The dataset's version used in this article integrates interactions curated from PubMed up to March 2021. The pan-viral query of PubMed used for the curation aims at identifying all peer-reviewed artciles which are likely to describe a physical interaction between a human and a viral protein of any kind. It has been optimized through the years and following benchmarks of public datasets. Every 3 months, a set of papers is extracted from PubMed for examination of their abstracts by our curators. More than 120,000 abstracts have been assessed so far. About 10 % of the papers are found susceptible to truly contain an interaction description. Then, selected articles are analyzed in depth. The dataset contains ca. 5,000 articles which were fully validated as they contained at least one interaction description that fulfills the following criteria (based on the PSI-MI recommendations(2):

1. The article must be peer-reviewed.
2. Reviews without experimental evidence are discarded.
3. The article identifier is a PMID.

4. The host protein must be human and wild type. It often requires checking the nature of cell lines, the description of plasmids, the origin of cDNA libraries, the alignment of primers with the gene and so on. If no evidence, the article is discarded.
5. The identifier of the host protein is from Swiss-Prot human non-redundant reference proteome(3).
6. The taxon of the viral protein must be clearly identified, and the protein must be wild type. Strain is annotated when clearly described, if not the strain of reference is used for the viral protein.
7. The viral protein is identified in UniProt(4) and described with a standard generic name. Mature proteins are annotated from polyproteins (see below for more details).
8. The method should detect physical interactions. For example, subcellular colocalization, functional interaction, reference to previously demonstrated interaction are excluded. However, in addition to binary methods (e.g., co-crystallization or yeast two-hybrid) methods allowing identification of complexes, such as co-immunoprecipitations, are also accepted.
9. The method is identified in the PSIMI controlled vocabulary dedicated to interaction detection methods(2). This allows filtering of methods and building of golden and binary datasets.
10. When available, sequences of interaction domains on each protein are collected. They should be clearly identified in the article, and they must perfectly align with at least one isoform of the source protein.

Regular benchmarks of public datasets systematically lead to a manual (re)curation of all the articles that may have been missed or misinterpreted in the curation process. Altogether, every paper identified in Vinland has been manually examined and validated, sometimes twice.

Regarding high-throughput papers, the rawest data available (e.g., ORF sequences) are assessed through alignment procedures in order to check the identity of interactors and assign a Swiss-Prot or a UniProt accession number. This often improves the quality of such high-throughput curated datasets.

A special attention is brought on viral protein annotations:

1. By giving a generic name to each viral protein. For example, UniProt entries describing a sequence for a NS1 protein of influenza viruses are annotated with names such as “non structural protein”, “non structural protein 1”, “non-structural protein 1”, “NS-1”, “NS1”. In our curation process these entries are assigned with a generic, standard name = “NS1”.

2. By finely annotating mature viral proteins. Viruses such as Dengue virus encode a polyprotein which is cleaved into mature proteins. In protein sequence databases, mature proteins rarely have specific accession numbers or are not even described in the processing section as a mature entity of the polyprotein. Therefore, in interactome public databases interactions with Dengue proteins are not correctly annotated. The polyprotein is often reported as being the only partner without more details. To overcome this, a specific entry is created for the viral mature protein. It is composed of the accession number of the polyprotein associated with start and stop positions coordinates of the mature protein. For example, Dengue Virus 1 NS5 in the B5AGU1 polyprotein becomes B5AGU1[2494-3392].

Those unique layers of annotation improve query and representation capacities of virus-host interactomes in Vinland. For example, users can retrieve interactions of all the NS5A proteins of HCV as well as graphically represent a clean interactome of a given viral species where one viral mature protein is shown as one node.

Such a massive curation effort requires a curation database, distinct from Vinland and called Drakkar. This provides assistance to curators and ensures data consistency. Drakkar is a PostgreSQL base accompanied by a PHP interface and a feature-rich form built with ReactJS to insert new protein-protein interaction descriptions. Drakkar stores the whole curation history:

- It is periodically fed with batches of articles. Curators can review pending publications and select or discard them with comments. Keywords are highlighted in abstracts.
- For selected publications, curators have access to a highly specific web form to annotate protein-protein interactions. The guaranties of this form are that it enables:
    - To track the PMID
    - To choose an existing interaction detection method (PSIMI identifier) with assistance
    - To choose a Swiss-Prot accession number for the human protein with assistance
    - To choose a UniProt accession number for the viral protein with assistance
    - If necessary, to define or reuse a mature viral protein in a UniProt polyprotein entry
    - To choose or reuse from the existing catalog a generic name for the viral protein
    - To align interaction mapping sequences on all sequences available for the source protein and confirm final associations.
- When the form is filled out, the curator can confirm the creation of the interaction description. A random stable Vinland id and a version number are thus assigned after non redundancy check. It is always possible to delete or update an interaction description in Drakkar, which uses soft-delete feature, timestamp and incremented version number.
- Once a paper has been completely finalized, the curator flags it as “curated”.

Drakkar has been designed to manage protein entries from different Uniprot/ Swiss-Prot releases. It keeps track of the most recent version of protein entries in such a way that new interaction descriptions added through the web form can only reference those entries. It also facilitates the manual review of interactor identifiers assigned on previous protein releases when necessary, i.e., when entries have been deleted or modified.

When integrating a new protein entry release of UniProt/Swiss-Prot into Drakkar, are only added:

- New protein entries which have accession numbers so far unknown
- Entries with known accession numbers but with updated data (primary name, sequence or taxon id) compared to the previous release used.

Protein entries from the last UniProt/Swiss-Prot release are flagged to be the only ones listed in the form. Consequently, as stated above, new interaction descriptions can only reference them. Furthermore, older interaction descriptions referencing now unflagged protein entries (modified or deleted in the last release) can easily be listed for manual reviewing, which may lead to modifications of interactor identifiers.

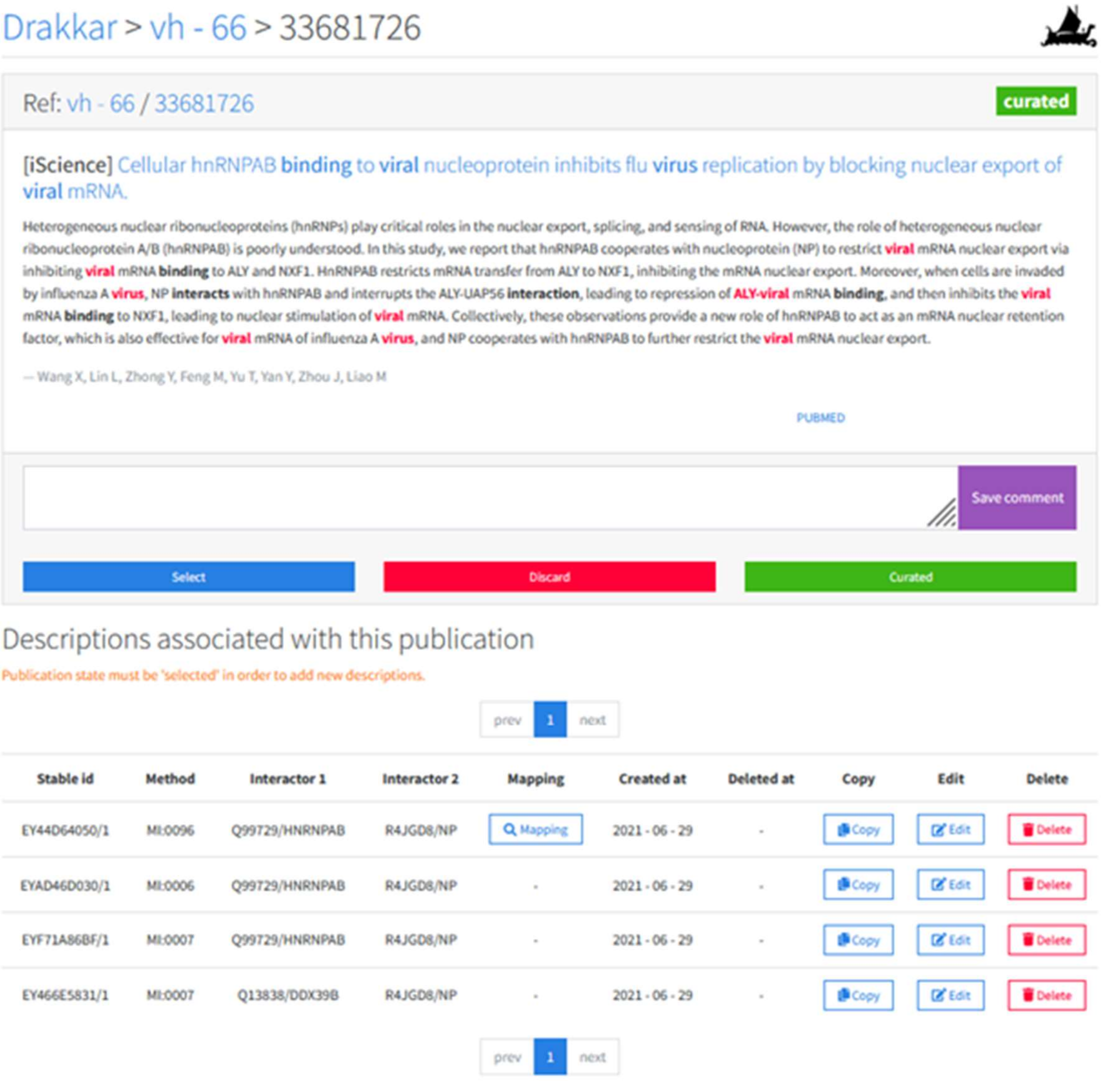

Drakkar > vh - 66 > 33681726

Ref: vh - 66 / 33681726 curated

[iScience] Cellular hnRNPAB binding to viral nucleoprotein inhibits flu virus replication by blocking nuclear export of viral mRNA.

Heterogeneous nuclear ribonucleoproteins (hnRNPs) play critical roles in the nuclear export, splicing, and sensing of RNA. However, the role of heterogeneous nuclear ribonucleoprotein A/B (hnRNPAB) is poorly understood. In this study, we report that hnRNPAB cooperates with nucleoprotein (NP) to restrict **viral** mRNA nuclear export via inhibiting **viral** mRNA **binding** to ALY and NXF1. HnRNPAB restricts mRNA transfer from ALY to NXF1, inhibiting the mRNA nuclear export. Moreover, when cells are invaded by influenza A **virus**, NP **interacts** with hnRNPAB and interrupts the ALY-UAP56 **interaction**, leading to repression of **ALY-viral** mRNA **binding**, and then inhibits the **viral** mRNA **binding** to NXF1, leading to nuclear stimulation of **viral** mRNA. Collectively, these observations provide a new role of hnRNPAB to act as an mRNA nuclear retention factor, which is also effective for **viral** mRNA of influenza A **virus**, and NP cooperates with hnRNPAB to further restrict the **viral** mRNA nuclear export.

— Wang X, Lin L, Zhong Y, Feng M, Yu T, Yan Y, Zhou J, Liao M

PUBMED

Save comment

Select | Discard | Curated

Descriptions associated with this publication

Publication state must be 'selected' in order to add new descriptions.

prev 1 next

Stable id	Method	Interactor 1	Interactor 2	Mapping	Created at	Deleted at	Copy	Edit	Delete
EY44D64050/1	MI:0096	Q99729/HNRNPAB	R4JGD8/NP	Mapping	2021 - 06 - 29	-	Copy	Edit	Delete
EYAD46D030/1	MI:0006	Q99729/HNRNPAB	R4JGD8/NP	-	2021 - 06 - 29	-	Copy	Edit	Delete
EYF71A86BF/1	MI:0007	Q99729/HNRNPAB	R4JGD8/NP	-	2021 - 06 - 29	-	Copy	Edit	Delete
EY466E5831/1	MI:0007	Q13838/DDX39B	R4JGD8/NP	-	2021 - 06 - 29	-	Copy	Edit	Delete

prev 1 next

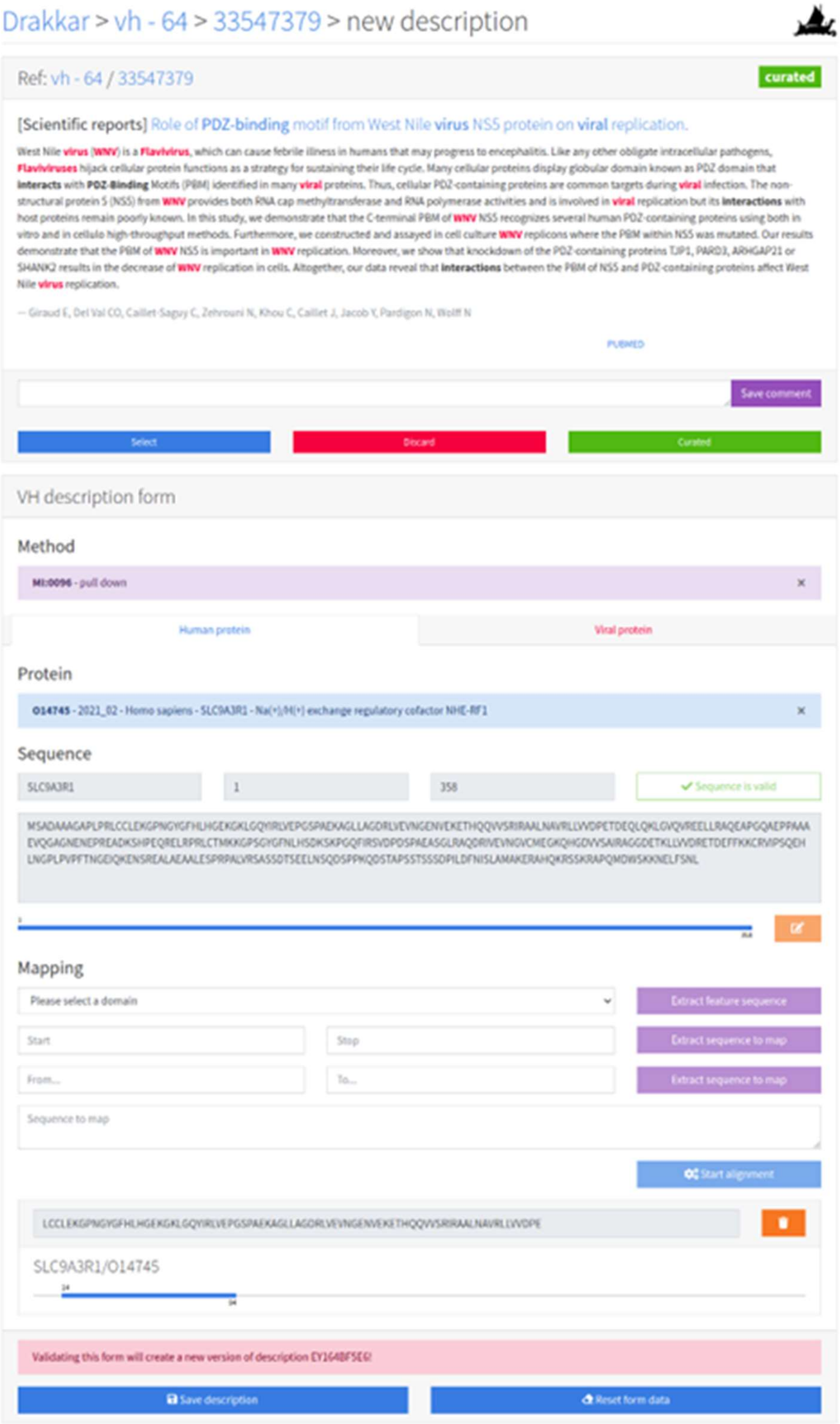
Drakkar > vh - 64 > 33547379 > new description
Ref: vh - 64 / 33547379
curated
[Scientific reports] Role of PDZ-binding motif from West Nile virus NS5 protein on viral replication.
West Nile virus (WNV) is a Flavivirus, which can cause febrile illness in humans that may progress to encephalitis. Like any other obligate intracellular pathogens, Flaviviruses hijack cellular protein functions as a strategy for sustaining their life cycle. Many cellular proteins display globular domain known as PDZ domain that interacts with PDZ-Binding Motifs (PBM) identified in many viral proteins. Thus, cellular PDZ-containing proteins are common targets during viral infection. The non-structural protein 5 (NS5) from WNV provides both RNA cap methyltransferase and RNA polymerase activities and is involved in viral replication but its interactions with host proteins remain poorly known. In this study, we demonstrate that the C-terminal PBM of WNV NS5 recognizes several human PDZ-containing proteins using both in vitro and in cellulo high-throughput methods. Furthermore, we constructed and assayed in cell culture WNV replicons where the PBM within NS5 was mutated. Our results demonstrate that the PBM of WNV NS5 is important in WNV replication. Moreover, we show that knockdown of the PDZ-containing proteins TJP1, PARD3, ARHGAP21 or SHANK2 results in the decrease of WNV replication in cells. Altogether, our data reveal that interactions between the PBM of NS5 and PDZ-containing proteins affect West Nile virus replication.
— Giraud E, Del Val CO, Caillet-Saguy C, Zehrouni N, Khou C, Caillet J, Jacob Y, Pardigon N, Wolff N
PUBMED
Save comment
Select
Discard
Curated
VH description form
Method
MI:0096 - pull down
Human protein
Viral protein
Protein
O14745 - 2021_02 - Homo sapiens - SLC9A3R1 - Na(+)/H(+) exchange regulatory cofactor NHE-RF1
Sequence
SLC9A3R1
1
358
Sequence is valid
Mapping
Please select a domain
Extract feature sequence
Start
Stop
Extract sequence to map
From...
To...
Extract sequence to map
Sequence to map
Start alignment
SLC9A3R1/O14745
Validating this form will create a new version of description EY164BF5E6!
Save description
Reset form data

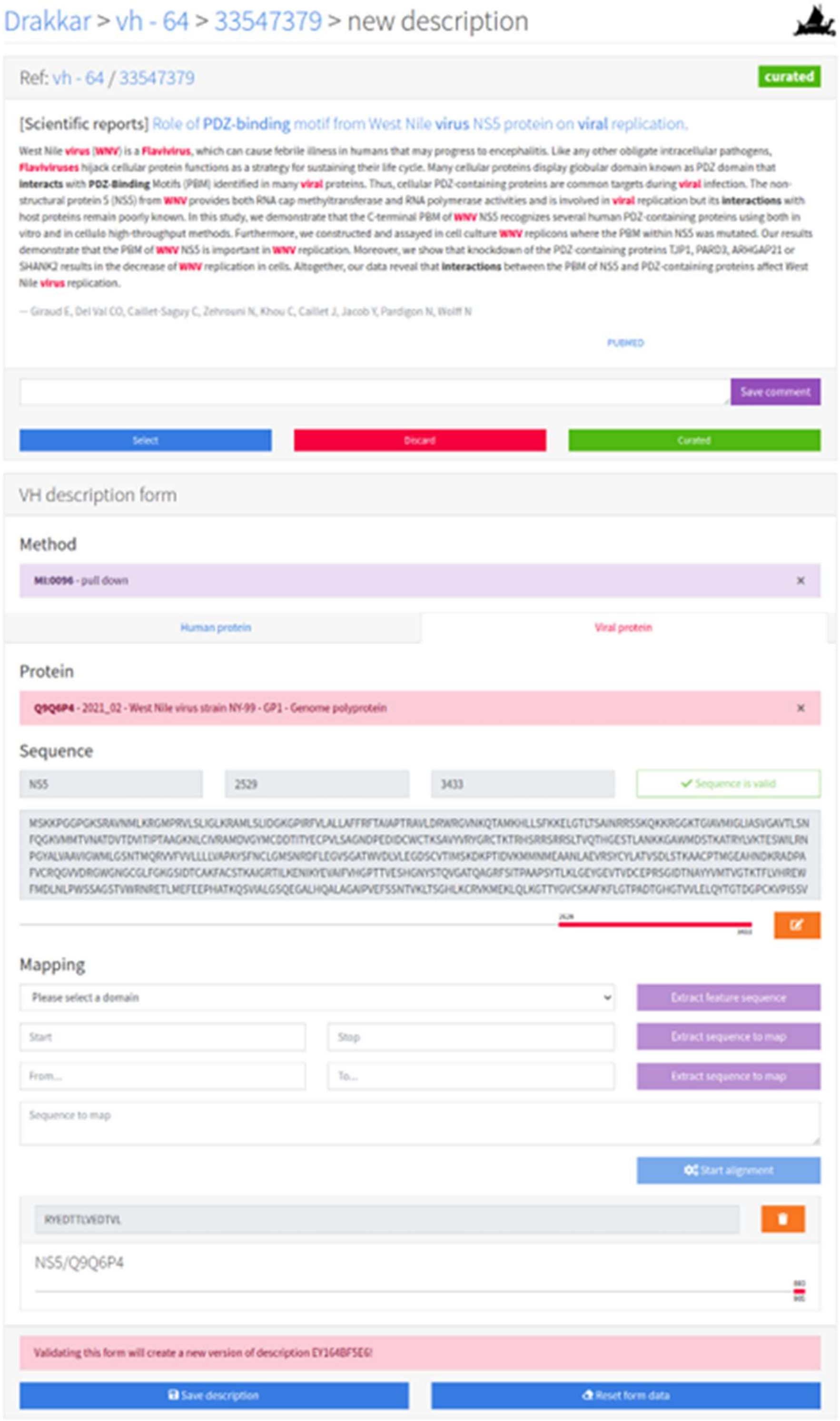
Drakkar > vh - 64 > 33547379 > new description
Ref: vh - 64 / 33547379
curated
[Scientific reports] Role of PDZ-binding motif from West Nile virus NS5 protein on viral replication.
West Nile virus (WNV) is a Flavivirus, which can cause febrile illness in humans that may progress to encephalitis. Like any other obligate intracellular pathogens, Flaviviruses hijack cellular protein functions as a strategy for sustaining their life cycle. Many cellular proteins display globular domain known as PDZ domain that interacts with PDZ-Binding Motifs (PBM) identified in many viral proteins. Thus, cellular PDZ-containing proteins are common targets during viral infection. The non-structural protein 5 (NS5) from WNV provides both RNA cap methyltransferase and RNA polymerase activities and is involved in viral replication but its interactions with host proteins remain poorly known. In this study, we demonstrate that the C-terminal PBM of WNV NS5 recognizes several human PDZ-containing proteins using both in vitro and in cellulo high-throughput methods. Furthermore, we constructed and assayed in cell culture WNV replicons where the PBM within NS5 was mutated. Our results demonstrate that the PBM of WNV NS5 is important in WNV replication. Moreover, we show that knockdown of the PDZ-containing proteins TJP1, PARD3, ARHGAP21 or SHANK2 results in the decrease of WNV replication in cells. Altogether, our data reveal that interactions between the PBM of NS5 and PDZ-containing proteins affect West Nile virus replication.
— Giraud E, Del Val CO, Caillet-Saguy C, Zehrouni N, Khou C, Caillet J, Jacob Y, Pardigon N, Wolff N
PUBMED
Save comment
Select
Discard
Curated
VH description form
Method
MI:0096 - pull down
Human protein
Viral protein
Protein
Q9Q6P4 - 2021_02 - West Nile virus strain NY-99 - GP1 - Genome polyprotein
Sequence
NS5
2529
3433
Sequence is valid
Mapping
Please select a domain
Extract feature sequence
Start
Stop
Extract sequence to map
From...
To...
Extract sequence to map
Sequence to map
Start alignment
RYEDTTLVEDTVL
NS5/Q9Q6P4
Validating this form will create a new version of description EY164BF5E6!
Save description
Reset form data

For a new Vinland release, curation batches need to be complete, and all valid descriptions must be based on a unique and recent UniProt/Swiss-Prot release. Data are extracted from Drakkar to create a new version of Vinland from scratch.

Vinland database also uses PostgreSQL as RDBMS. The backend is a PHP API providing data formatted as JSON and the frontend uses this API to display the data through a single page application built with ReactJS (https://reactjs.org/). The network viewer combines D3js (https://d3js.org/) for computing the node positions during layout processing and Konva (https://konvajs.org/) for high performance html canvas rendering.

Vinland backend and frontend are hosted on the VirHostNet cloud IT infrastructure at the PRABI bioinformatics core facility.

Human interactome

A manually curated human-human protein-protein interaction dataset composed of 445,211 interaction descriptions was built using data from neXtProt release 2021-11-19 and integrated into the Vinland interface.

neXtProt is a knowledge platform that integrates human protein data from various sources and allows users to explore it using semantic technologies (1). It combines human-human protein-protein interaction data extracted independently from the literature by the IntAct, neXtProt and ENYO Pharma curation teams. This dataset is updated at each neXtProt release, i.e. at least twice a year.

For each interaction, the PubMed identifier of the article is reported. For IntAct and ENYO Pharma data, a PSI-MI term indicates which method was used. The ENYO Pharma subset of human-human protein-protein interaction data also contains the positions of the interacting regions on the human protein sequences when this information is available.

The most recent neXtProt human-human protein-protein interaction dataset can be retrieved using the following script: interaction-data2csv.py . It runs the appropriate SPARQL query, parses the json results from the query, removes the symmetrical data et writes the results in csv format.

Benchmark of virus-human protein-protein interaction datasets

Public databases which are described to collect experimentally verified virus-host protein-protein interactions through literature curation or direct user submissions were selected:

- IntAct(5)
- VirHostNet(6)
- HVIdb(7) that gathers interactions from multiple data resources such as VirusMentha and PHISTO

Their datasets were downloaded in April 2021. Filters were applied to select interactions, described in PubMed articles, between one viral and one human protein. PMID identifiers were extracted from each dataset, making a single, non-redundant list per resource. Lists were compared with Vinland's articles.

Network metric computation

Protein-protein interaction networks are formed by a set of nodes (or vertices) representing proteins, connected by edges representing physical interactions between these proteins. The topology of protein-protein interaction networks can be described with a range of metrics. The degree or connectivity (k) of a node v in a graph is a local centrality measure summarizing the number of edges incident to this node v. The betweenness (b) of a node v in a graph is a global centrality measure which can be defined by the number of shortest paths going through this node and is normalized by twice the total number of protein pairs in the graph n*(n-1). Distributions were compared using Wilcoxon test.

Examples of viral strategies to hijack cellular functions

As viruses strictly depend on the host translation machinery to synthesize their proteins (8), GO terms related to "Translation" are unsurprisingly found among the most significantly enriched. For example, the influenza A NS1 multifunctional protein disrupts 3'-end processing of cellular mRNAs by binding CPSF30 (9). Conversely, the human cytomegalovirus UL38 protein interacts with TSC1 and TSC2 and antagonizes their ability to inhibit mTORC1 (10). Thereby, UL38 maintains the activity of mTORC1 and promotes cap-dependent translation of selective host factors (11). This illustrates that viruses provide highly diverse clues on how to reprogram the translational capacity of the cell. The transport to the nucleus is also highly targeted by viruses, and KPNA2 is the most targeted protein in the current infection landscape (Vinland). For instance, both influenza viruses and herpesvirus saimiri have evolved a protein that promotes viral RNA transport through the nuclear pore complex(12),(13). Influenza virus NP and herpesvirus saimiri ORF57 bind KPNA2 using a nuclear localization signal enriched in basic residues mimicking human NLS. Despite a probable inspection bias towards this biological process in the literature, the targeting of some human proteins by multiple viruses not only indicates the crucial importance of these targets in the virus cycle but also shows that parallel strategies were developed by unrelated viruses to manipulate the function of essential proteins.

Examples of information generated by viral protein interfaces with human proteins

This level of annotation allows the anticipation of the local human protein network perturbation that could arise from the expression of a viral protein. For instance, PIK3R1 is potentially able to interact with numerous differential human proteins through its SH3(14) or SH2 domains(15),(16) (Figure below). Some viral proteins such as HCV NS5A(17) or HEV Orf3(18) have evolved similar strategies to target PIK3R1 SH3, while others such as γ-herpesvirus M2(19) or HIV1 Nef(20) have developed the ability to target PIK3R1 SH2. Both viral protein groups do not compete with the same set of human proteins to bind PIK3R1.

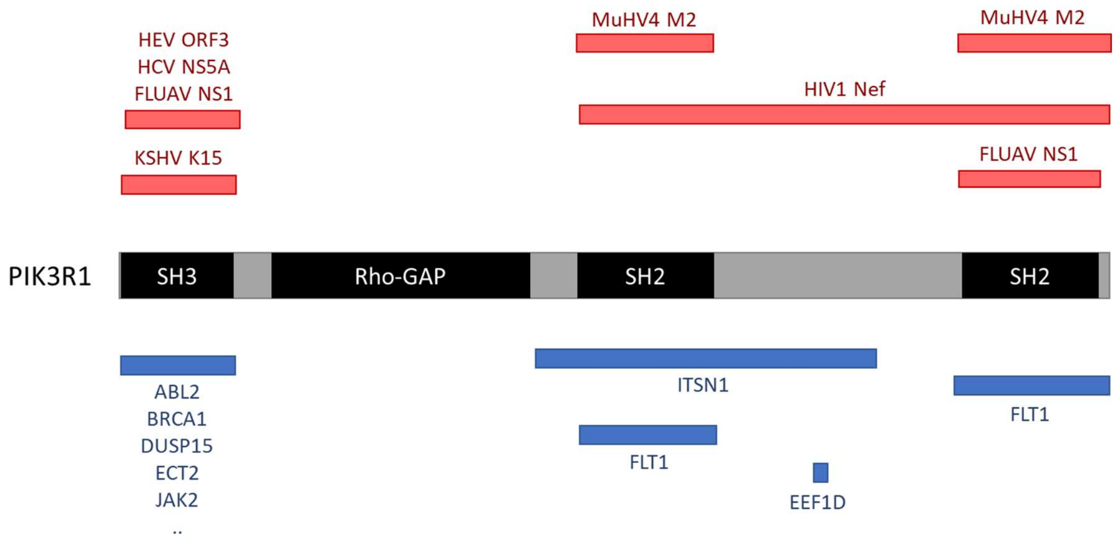

PIK3R1 interactome. *PIK3R1 is represented with its structural domains. Regions of PIK3R1 interacting with viral proteins and a selection of human proteins are shown in red and blue, respectively.*

Identification of known SLiMs in viral peptide sequences

List of ELM classes were downloaded from Eukaryotic Linear Motif resource(21) and regex were compared to viral peptide sequences of Vinland.

Identification of drugged targets of viral peptides

Targets of FDA-approved drugs were downloaded from DrugBank Drug Targets Dataset of Harmonizome (22) and compared with targets of the viral peptide library.

Examples of short linear motifs (SLiMs) identified on viral proteins

PDZ binding motifs are identified on the C-terminus of many viral proteins(23). PDZ is a modular domain found on proteins that organize a large diversity of cellular functions(24). Other SLiMs are specific to a single human protein. For example, Rotavirus A NSP1 protein interacts with IRF3, mimicking the eukaryotic motif pLxIS (p represents any hydrophilic residue, x represents any residue) to subvert the innate immune response. Interestingly, this motif can be found in the sequence of other IRF3 viral interactors, such as in vIRF1 of KSHV(25) or in nsp16 of SARS-CoV(26). While the studies describing these interactions did not identify the motif, it could be responsible for the binding of vIRF1 and nsp16 to IRF3.

Viral peptide library

The library of peptides was synthesized at Proteogenix, Schiltigheim, France, with a purity grade above 70 % and solubilized in PBS at 10 mM.

Peptide activity graphs

GraphPad Prism was used to represent peptide activities and analyze individual dose-response curves with a non-linear regression model.

Results of the peptide repositioning screening approach for several pathogens

Contrary to influenza viruses, the HCV (group IV, (+) single-stranded RNA virus) can establish chronic infections. The library was screened on HCV replication in Huh7 cells, using a subgenomic HCV replicon expressing the firefly luciferase whose activity is related to replicon RNA copy number in the cell. The impact of peptides on HIV1 (group VI, retrovirus) replication was monitored in HeLa P4 cells carrying a Tat-inducible LacZ gene 48 hr post-infection by measuring β-galactosidase activity. Finally, for EBV (group I, double-stranded DNA virus), chemical induction of productive EBV cycle in the presence of peptides was monitored using a luciferase reporter gene cloned downstream of the BdRF1 promoter, in a plasmid stably transfected in Hone1 cells. BdRF1 is a late gene in the EBV cycle. Luciferase activity was measured 48 hr post-treatment. For instance, for EBV **(Figure 3B)**, CPEP95 (identified on E2 protein of HPV16) targets TP53(27) and inhibits the signal by 60 %. This is consistent with a recent study suggesting that EBV reactivation by HIF1α requires TP53(28). CPEP116 identified on HIV1 Gag protein(29) targets TSG101 whose depletion impairs promoter transactivation of five Rta-responsive EBV late genes(30). Conversely, CPEP1 (from HHV3 IE4(31)) and CPEP71 (from HIV1 Integrase(32)) enhance EBV signal by more than 2-fold. These peptides respectively target SRSF3 and IPO7 that are described to be restriction factors for the virus(33,34)..

The most potent anti-HIV1 peptide **(Figure 3C)** (CPEP77 from HHV8 vIRF4(35)) targets the deubiquitinase USP7, a protein described to stabilize Tat and enhance virus production(36).

CPEP103 (from HCV NS4A(37)) targeting EEF1A1 is also consistent with HIV1. EEF1A1 interaction with the reverse transcriptase is required for HIV reverse transcription and replication(38). CPEP72 (from human RSV fusion protein(39)) interacts with RHOA and strongly enhances the beta-galactosidase signal in the HIV1 screen. RHOA signal transduction pathway is known to inhibit HIV1 replication(40). Similar data are obtained for CPEP2 (from Vaccinia virus F1(41)) which interacts with Caspase 9 and enhances the signal by almost 9-fold. Accordingly, inhibiting caspases stimulates HIV1 replication(42).

Strikingly, 5 out of the 10 best HCV enhancer peptides **(Figure 3D)** are also among the top enhancers of HIV1 signal in the screens, including peptides targeting CASP9 and RHOA. As seen with HIV1, inhibiting RHOA expression by siRNA increases HCV replication(43). CPEP144 is a peptide inhibiting HCV that originates from EBV LMP1, a protein described to activate IFN regulatory factor 7 (IRF7)(44). It is tempting to anticipate that CPEP144 might counteract HCV NS5A suppression of IRF7-mediated activation of IFNα promoters(45). Finally, inhibition of RNA replicon levels with CPEP145 targeting KPNA2 (from HPV16 L2(46)) is reminiscent of the inhibition of HCV RNA levels induced by nuclear localization signal peptides(47).

Incorporation of propidium iodide (PI) in F. novicida-infected bone marrow-derived macrophages (BMDM) was measured in real time during 20 hr, in the presence or not of peptides(48). Francisella enters cells by phagocytosis to be incorporated in Francisella-containing phagosome (FCP)(49). FCP matures until the late endosome stage. The FCP membranes are disrupted in an acidification-dependent manner leading to the release of bacteria into the cytosol where they replicate. Proliferation is followed by the progressive death of macrophages. Interestingly, some of the most active peptides to inhibit PI accumulation following infection target proteins localized at the phagosome during phagocytosis **(Figure 3E)**. Among them, CPEP113 (from HCV NS5B(50)) interacts with NCL which is reported to promote Francisella tularensis infection and colocalize with the bacteria until bacterial release in the cytosol(51). PDLIM2, another critical protein for phagocytosis in macrophages(52) is the target of CPEP59 (from influenza A virus NS1(53)) which is one of the most potent virus-derived peptides to inhibit PI incorporation.

Complement to CPEP31 translation into small organic molecule

Despite an increased interest in peptides as therapeutics, their progression to clinical development faces several challenges(54) including oral bioavailability, stability and resistance to serum proteases, ability to cross the membrane as well as manufacturing costs. Some of these hurdles can be overcome by translating non-sequential putative peptide hotspots into a library of small molecules **(Figure 1A and 1B)**. α-helices structural features are considered favorable for this kind of translation(55). For a proof of concept, a virtual screening approach which aims at finding small organic molecules able to mimic the CPEP31 peptide should rely solely on its own structural information and may not involve prior knowledge of potential protein targets.

In the full structure of vFLIP, the surface of the peptide is not accessible to the solvent and its folding may be influenced by the rest of the protein. The search for 3D structures of analogous sequences using blast yielded examples with very similar folding (PDB: 3TSV and 2V8I), as shown in the figure below.

3cl3	E	V	V	L	F	L	L	N	V	F
	h	h	h	h	h	h	l	l	l	l
3tsv	E	A	V	L	F	L	L	D		
	h	h	h	h	h	h	h	h		
2v8i				L	F	L	L	N	V	
				h	h	h	l	l	l	

Alignment of the sequence of peptide CPEP31 (top line) with analogous sequences for which a protein 3D structure exists in the Protein Data Bank. The nature of the secondary structure observed is indicated below each sequence; h (orange): alpha-helix, l (yellow): loop.

Moreover, in these structures, the analogous peptide sequences are accessible to the solvent, suggesting that the influence of the rest of the structure on the folding of CPEP31 in 3CL3 can be ignored.

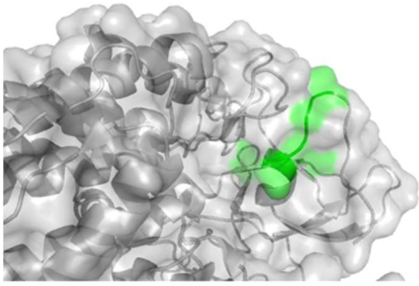

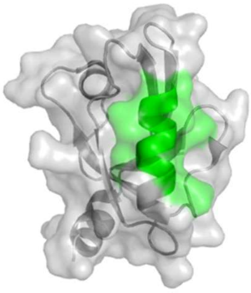

Left: Structure of PDB entry 2V8I with sequence LFLLLNV highlighted in green. This short sequence forms an alpha helix and a loop and is present on the surface of the protein. Right: structure of PDB entry 3TSV with sequence EAVLFLLD highlighted in green, forming an alpha helix at the surface of the protein.

Therefore, the structure observed in 3CL3 is used as an approximation of the vFLIP peptide alone in solution.

As the full peptide has a much larger volume than an organic molecule complying with the Lipinski rule of five (as shown for CPEP31 in **Figure 4B**), the approach consisted in using the structure of some key amino acid residues and searching for small organic molecules which are similar in shape. The three amino acids LFL in the middle of CPEP31 are proposed to be a hotspot for the peptide's activity on the protein ATG3(56), since a loss of activity in a cellular assay is observed for

a mutant peptide where all three residues are mutated at the same time. However, because of their central position, their mutation is likely to affect the complete structure and the observed loss of activity may not reflect the direct involvement of these residues with the protein target.
Filters applied on the top 0.5% scoring molecules: First, in order to be considered a hit, the shape of the molecule must cover the shape of all three residues used to build the query. In addition, it should not display an obvious polar mismatch such as having a highly polar or charged group near a hydrophobic feature of the query. Then, structures with more than 6 rotatable bonds, deemed too flexible, as well as molecules judged too simple (measured by the size of their functional-class fingerprint(57)), were removed from further consideration. Molecules having three better scoring analogs among the hits were also excluded. For this step, the similarity between two molecules was measured with the Tanimoto coefficient on the same fingerprint.

Kinetic solubility

10 mM DMSO Stock solutions of tested compounds were distributed into filtration plate (Multiscreen Durapore 0.45µm) containing PBS pH 7.4 buffer to a final maximum concentration of 130 µM (1.3 % DMSO/buffer). Solutions or suspensions were shaken for 30 min at room temperature. The samples were filtered under vacuum into a collection plate and diluted 50 % with ACN for UV analysis. Quantification was performed against a Reference plate containing the same tested compounds fully solubilized in ACN/PBS buffer 1:1. Reference and Sample plates were analyzed by UPLC/UV-MS using a generic UPLC method. The measured solubilities are presented as mean values of tetraplicate determinations, with a maximum solubility threshold of 100 µM, and the lower limit of quantitation governed by the UV absorption properties of the compound.

Microsomal stability

Tested compounds (1 µM) were incubated with pooled human liver microsomes (Xenotech; pooled male and female) and pooled mouse liver microsomes (Xenotech; male CD-1 mice). Microsomes (final protein concentrations of 0.25 and 0.5 mg/ml, 0.1 M phosphate buffer pH 7.4) containing tested compound (final substrate concentration 1 µM; final DMSO concentration 0.25 %) were preincubated at 37 °C prior to the addition of NADPH (final concentration 1 mM) to initiate the reaction. The final incubation volume was 200 µl. 3 species-specific control compounds were included with each species. At 5 time points (0, 5, 10, 20, and 40 min), reactions were stopped by transferring 50 µl of the incubation mixture into 0.5 µM carbamazepine in acetonitrile. Tested compound concentrations were analyzed by LC-MS/MS and the resulting data were used to determine the half-life and intrinsic clearance of the compound in each species.

Cryopreserved hepatocyte stability

Tested compounds (1 µM) were incubated with human cryopreserved hepatocytes (pool of 5) (IVT Celsis) and mouse cryopreserved hepatocytes (IVT Celsis). Hepatocytes (1, 0.5 or 0.25 x10^6 cells/mL, Williams' Medium E (WME) with 4 mM L-glutamine and 2 mM magnesium sulphate) containing tested compound (final substrate concentration 1 µM; final DMSO concentration 0.25 %) were preincubated at 37 °C. The final incubation volume was 500 µl. 3 species-specific control compounds were included with each species. At 7 time points (0, 10, 20, 40, 60, 90, and 120 min), reactions were stopped by transferring 50 µl of the incubation mixture into 0.5 µM carbamazepine in cold acetonitrile. Tested compound concentrations were analyzed by LC-MS/MS and the resulting data were used to determine the half-life and intrinsic clearance of the compound in each species.

Plasma protein binding

The free and bound fractions of the tested compounds in mouse and human plasma were determined by equilibrium dialysis using a 96-well Teflon dialysis unit (HTDialysis plate complete unit, HT Dialysis, USA). The HTDialysis plate complete unit consists of a 96-well plate with wells vertically bisected by a semipermeable membrane (membranes strips 12 to 14 kDa) creating two chambers. A 125 µL aliquot of plasma containing tested compound at 10 µM was placed on one side and 125 µL of Dulbecco's phosphate buffered saline (D-PBS) on the other. The plate was incubated at 37 °C for 6 hr under gentle agitation. Then, samples from both plasma and buffer compartment were analyzed by a LC-MS/MS method to measure the free fraction and the bound fraction to the plasma proteins.

PK parameters and tissue distribution in mice

The pharmacokinetic profiles of the tested compounds were evaluated in male Swiss (CD-1) mice around 5-6 weeks old (3 animals per sampling time). The compound was administered either i.v. at 0.25 mg/kg, formulated as a 0.05 mg/mL solution in 10 % DMSO, 50 % PEG200, 40 % water or p.o. at 2 and 20 mg/kg, formulated as a 0.4 and 4 mg/ml solution in 1.5 % CMC, 0.2 % Tween 80 in water. Blood samples were collected (0.25 mL) from the sinus retro-orbital vein at 0.03, 0.08, 0.25, 0.5, 1, 2, 4, 8, and 24 hr for i.v. route and 0.08, 0.25, 0.5, 1, 2, 4, 8, and 24 hr for p.o. route. The blood samples were immediately centrifuged at 2500 rpm at +4 °C, and the plasma was stored at −80 °C. 50 µL of plasma sample was taken and 150 µL of acetonitrile was added. After protein precipitation, analysis of the molecule was performed using LC-MS/MS to determine plasma compound concentration. For liver and lung collection, 3 animals per group were involved and sampled after 2 hr and 24 hr upon 20 mg/kg p.o. administration. Animals were perfused with around 10 mL of saline solution directly into the heart to extract the maximal blood sample from the liver and the lung. Animals were then sacrificed by cervical dislocation and liver

and lung were collected. Liver and lung were precisely weighed and were stored frozen at target temperature of -80 °C.

Complement to hit optimization

Analogs on the 3-carboxamide part of dEF384 did not lead to significant improvement in potency. However, the presence of the carboxylic acid on the 2-amido group of various derivatives proved to be important for maintaining the initial activity with a favorable mouse microsomal stability (Clint<20 µl/min/µg for dEF384) and very good PK properties for dEF384 and its analogs **(Figure 5B)**.

NASH histopathology and immunochemistry

Livers were fixed in 10 % neutral buffered formalin and paraffin-embedded; and sections were stained with H&E and picrosirius red to analyze their morphology. Histopathological analysis was performed by a pathologist blinded to the study. Fibrosis was evaluated by quantification of the picrosirius red positive areas. Inflammation was graded by counting the H&E positive inflammatory foci per field.

Kidneys were fixed in 4 % PFA and paraffin embedded. 4-µm sections were stained with picrosirius red. Images were acquired using a digital slide scanner (Nanozoomer S210 Digital Slide Scanner, Hamamatsu). Renal lesions were blindly examined by a renal pathologist. The extent of interstitial fibrosis was quantified using Image J software (NIH) on Sirius red scanned colored sections. In short, at least 20 random selected fields (original magnification, ×200) across the kidney cortex were analyzed per each kidney section, and the results were expressed as percentages of the total area of the selected fields.

For immunohistochemistry, 4-µm sections of paraffin-embedded kidneys were submitted for appropriate antigen retrieval by high pressure heating in a solution of Tris EDTA Tween or in a citrate solution. Then, sections were then incubated overnight at 4°C with the following antibodies: rabbit monoclonal anti-CD3 (Abcam, 1:100) and rat monoclonal anti- F4/80 (Biorad, 1:100). After washing, sections were incubated with appropriate amplification secondary antibody: biotin-coupled anti-rabbit (GE Healthcare) for CD3 and biotin-coupled anti-rat (Vector Laboratories) for F4/80, and then incubated with streptavidin coupled HRP (Southern). At the end, DAB (DAKO) staining was used to detect HRP. For quantification of CD3+ and F4/80+ renal cells, the degrees of cellular infiltrate were automatically quantified using ImageJ software (NIH) and expressed as a percentage of the surface area of the positive staining per field, in at least 10 random fields perp kidney section.

Supplementary Figure

Fig. S1. Summary of the virtual screening process

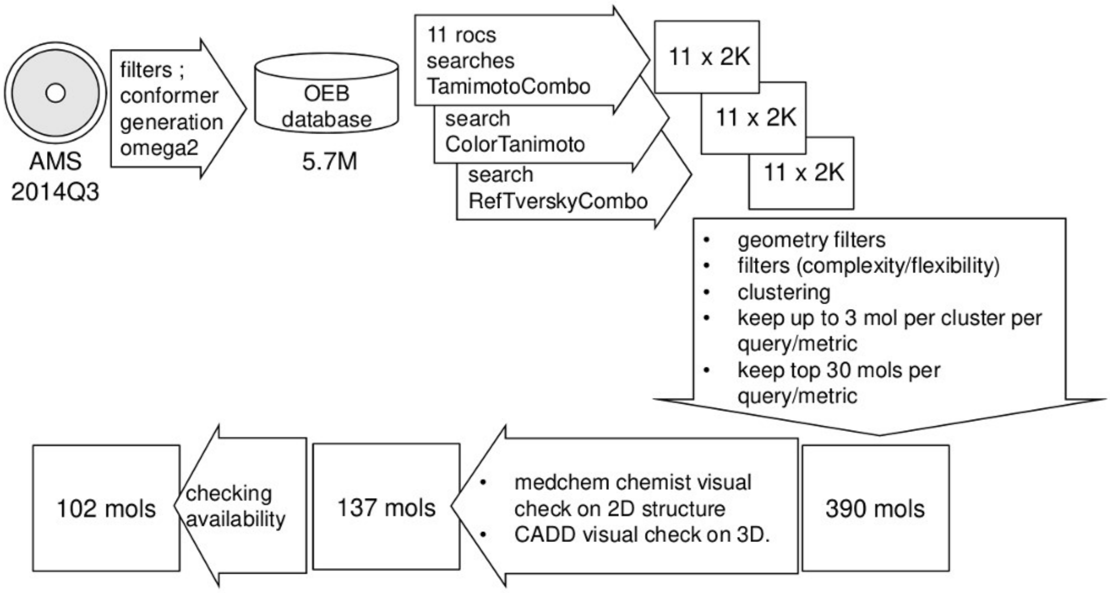

Supplementary Tables

Table S1. Description of the library of the viral peptides synthesized (in fusion with a HIV1 Tat protein cell-penetrating sequence) and screened in several cell-based assays. Each peptide is described with its sequence, its source (protein accession name and taxon), its target(s) (protein accession name and taxon) and the PMID of the publication where it has been originally described.

peptide ID	peptide sequence	source accession	source name	source ncbi taxon id	source taxon	target accession	target name	target ncbi taxon id	target taxon	pmid
CPEP131	LEDLL	Q77AI1	E7	333760	Human papillomavirus type 16	P20226	TBP	9606	Homo sapiens	9349482
CPEP131	LEDLL	Q77AI1	E7	333760	Human papillomavirus type 16	P17936	IGFBP3	9606	Homo sapiens	10938125
CPEP130	LQQLL	Q77YF9	Vpr	11676	Human immunodeficiency virus 1	P37231	PPARG	9606	Homo sapiens	17932108
CPEP152	LHCYE	G5CD65	E7	10585	human papillomavirus 31	P06400	RB1	9606	Homo sapiens	15016876
CPEP152	LHCYE	P17387	E7	10585	human papillomavirus 31	O60934	NBN	9606	Homo sapiens	24850735
CPEP21	YASIL	Q9QR69	K15	868565	Human herpesvirus 8 strain GK18	O00165	HAX1	9606	Homo sapiens	11752170
CPEP44	PVQES	Q98325	vFLIP	10280	Molluscum contagiosum virus subtype 1	Q13114	TRAF3	9606	Homo sapiens	16410799
CPEP33	HGCPL	Q14T47	NS5A	31647	Hepatitis C virus subtype 1b	O95292	VAPB	9606	Homo sapiens	16227268
CPEP132	PPPPY	Q1HVJ2	LMP2	82830	Epstein-barr virus strain ag876	Q96J02	ITCH	9606	Homo sapiens	11046148
CPEP132	PPPPY	Q1HVJ2	LMP2	82830	Epstein-barr virus strain ag876	P46934	NEDD4	9606	Homo sapiens	11046148
CPEP150	SKMQL	P03508	NS2	211044	Influenza A virus (A/Puerto Rico/8/1934(H	O14980	XPO1	9606	Homo sapiens	11118210
CPEP48	YRHHY	P69723	Vif	11706	HIV-1 M:B_HXB2R	Q9HC16	APOBEC3G	9606	Homo sapiens	19036809
CPEP48	YRHHY	P69723	Vif	11706	HIV-1 M:B_HXB2R	Q8IUX4	APOBEC3F	9606	Homo sapiens	17522216
CPEP154	YLTDM	P03496	NS1	211044	Influenza A virus (A/Puerto Rico/8/1934(H	O00459	PIK3R2	9606	Homo sapiens	16963558
CPEP54	PLDLS	P03255	E1A	28285	Human adenovirus 5	Q13363	CTBP1	9606	Homo sapiens	23864635
CPEP54	PLDLS	Q69140	EBNA6	82830	Epstein-barr virus strain ag876	Q13363	CTBP1	9606	Homo sapiens	11462050
CPEP70	RRKAK	B9VIK5	IN	11676	Human immunodeficiency virus 1	O95373	IPO7	9606	Homo sapiens	17360709
CPEP12	LTMVI	Q05128	VP40	128952	Ebola virus - Mayinga, Zaire, 1976	P53992	SEC24C	9606	Homo sapiens	18329616
CPEP12	LTMVI	Q05128	VP40	128952	Ebola virus - Mayinga, Zaire, 1976	P53992	SEC24C	9606	Homo sapiens	18329616
CPEP123	HVKNG	Q14T47	NS5A	31647	Hepatitis C virus subtype 1b	O95292	VAPB	9606	Homo sapiens	16227268
CPEP49	PTAPP	A1Z0U4	Gag	11676	Human immunodeficiency virus 1	Q99816	TSG101	9606	Homo sapiens	11427703
CPEP49	PTAPP	P04591	Gag	11706	HIV-1 M:B_HXB2R	Q99816	TSG101	9606	Homo sapiens	11427703
CPEP49	PTAPP	P18095	p6	11714	Human immunodeficiency virus type 2 (IS	Q99816	TSG101	9606	Homo sapiens	12388682
CPEP143	LNSLF	Q8USP9	Gag	57667	Simian-Human immunodeficiency virus	Q8WUM4	PDCD6IP	9606	Homo sapiens	21849430
CPEP58	PYQLT	P88961	vFLIP	435895	Human herpesvirus 8 type M	Q12933	TRAF2	9606	Homo sapiens	16311516
CPEP97	TYGTC	Q66346	prM	11060	Dengue virus 2	P36543	ATP6V1E1	9606	Homo sapiens	18573235
CPEP30	TGGER	Q6VGT0	E4-17K	28285	Human adenovirus 5	P29590	PML	9606	Homo sapiens	16501113
CPEP30	TGGER	Q6VGT0	E4-17K	28285	Human adenovirus 5	P29590	PML	9606	Homo sapiens	16501113
CPEP59	TIESEV	Q29SJ1	NS1	319102	Influenza A virus (A/chicken/Henan/12/200	Q96JY6	PDLIM2	9606	Homo sapiens	21625420
CPEP113	WRHRAR	D3GHM2	NS5B	11103	Hepacivirus C	P19338	NCL	9606	Homo sapiens	12427757
CPEP17	RRETQV	P06463	E6	333761	Human papillomavirus type 18	Q12959	DLG1	9606	Homo sapiens	17713926
CPEP35	HAGPIA	Q71B38	Gag	11676	Human immunodeficiency virus 1	P62937	PPIA	9606	Homo sapiens	9385632
CPEP68	EDPNEE	Q6VGW0	E1A	28285	Human adenovirus 5	Q09472	EP300	9606	Homo sapiens	12743606
CPEP26	PGYPWP	Q6UNT8	Core	11103	Hepacivirus C	P23458	JAK1	9606	Homo sapiens	12764155
CPEP26	PGYPWP	Q6UNT8	Core	11103	Hepacivirus C	O60674	JAK2	9606	Homo sapiens	12764155
CPEP18	DDPERE	P04601	Nef	11706	HIV-1 M:B_HXB2R	P04049	RAF1	9606	Homo sapiens	9624170
CPEP119	TGERDW	P69723	Vif	11706	HIV-1 M:B_HXB2R	Q8IUX4	APOBEC3F	9606	Homo sapiens	18619467
CPEP34	KALRRHR	F5HI84	UL37	10359	Human betaherpesvirus 5	Q07812	BAX	9606	Homo sapiens	17496930
CPEP56	PKKKRKV	P03070	LT	1891767	Macaca mulatta polyomavirus 1	O00410	IPO5	9606	Homo sapiens	25485706
CPEP32	SLQYLAL	P69723	Vif	11706	HIV-1 M:B_HXB2R	Q93034	CUL5	9606	Homo sapiens	15574592
CPEP151	PPIPPPR	Q7T4V8	NS5A	11103	Hepacivirus C	P62993	GRB2	9606	Homo sapiens	10318918
CPEP151	PPIPPPR	C5ISZ7	NS5A	11103	Hepacivirus C	P07948	LYN	9606	Homo sapiens	15784897
CPEP82	RKSASMS	Q9JG20	M	11171	Mumps virus Miyahara vaccine	P31946	YWHAB	9606	Homo sapiens	21147917
CPEP146	RHFPRIW	Q77YF9	Vpr	11676	Human immunodeficiency virus 1	P62937	PPIA	9606	Homo sapiens	20920334
CPEP117	AAAVAFL	P03247	E1B-55K	10515	Human adenovirus 2	O60238	BNIP3L	9606	Homo sapiens	10381623
CPEP76	FPESLIL	P03259	E1A	28282	Human adenovirus 12	Q92793	CREBBP	9606	Homo sapiens	10196247
CPEP148	RRRETQV	P06463	E6	333761	Human papillomavirus type 18	Q96QZ7	MAGI1	9606	Homo sapiens	17267502
CPEP148	RRRETQV	P06463	E6	333761	Human papillomavirus type 18	Q12959	DLG1	9606	Homo sapiens	24550280
CPEP148	RRRETQV	P06463	E6	333761	Human papillomavirus type 18	Q12959	DLG1	9606	Homo sapiens	17267502
CPEP148	RRRETQV	P06463	E6	333761	Human papillomavirus type 18	P63104	YWHAZ	9606	Homo sapiens	33723253
CPEP148	RRRETQV	P21735	E6	10593	human papillomavirus 45	Q12959	DLG1	9606	Homo sapiens	24550280
CPEP148	RRRETQV	P21735	E6	10593	human papillomavirus 45	Q14160	SCRIB	9606	Homo sapiens	24550280
CPEP75	KKTKSLP	P16629	M	11208	Simian virus 5 (strain W3)	P31946	YWHAB	9606	Homo sapiens	21147917
CPEP50	YFADLLI	P03304	2A	12104	Encephalomyocarditis virus	P06730	EIF4E	9606	Homo sapiens	21145089
CPEP24	TPQVPLR	P04601	Nef	11706	HIV-1 M:B_HXB2R	P06241	FYN	9606	Homo sapiens	8681387
CPEP111	DDLYEEV	Q9QR69	K15	868565	Human herpesvirus 8 strain GK18	P07948	LYN	9606	Homo sapiens	18985015
CPEP78	WKSKKTP	D3GHM2	NS5B	11103	Hepacivirus C	P19338	NCL	9606	Homo sapiens	12427757
CPEP145	LRKRRKRL	P03107	L2	333760	Human papillomavirus type 16	P52292	KPNA2	9606	Homo sapiens	15507604
CPEP144	PVQLSYYD	P03230	LMP1	10377	Human herpesvirus 4 strain B95-8	Q92985	IRF7	9606	Homo sapiens	19017798
CPEP108	GARASVLS	P04591	Gag	11706	HIV-1 M:B_HXB2R	P30419	NMT1	9606	Homo sapiens	18089753
CPEP108	GARASVLS	P04591	Gag	11706	HIV-1 M:B_HXB2R	O60551	NMT2	9606	Homo sapiens	18089753
CPEP4	QSSSSGSP	P03211	EBNA1	10377	Human herpesvirus 4 strain B95-8	P67870	CSNK2B	9606	Homo sapiens	20719947
CPEP134	GGKWSKLS	Q8Q621	Nef	11676	Human immunodeficiency virus 1	P30419	NMT1	9606	Homo sapiens	18089753

peptide ID	peptide sequence	source accession	source name	source ncbi taxon id	source taxon	target accession	target name	target ncbi taxon id	target taxon	pmid
CPEP134	GGKWSKLS	Q8Q621	Nef	11676	Human immunodeficiency virus 1	O60551	NMT2	9606	Homo sapiens	18089753
CPEP109	IKQEDIKP	P19893	IE2	10360	Human herpesvirus 5 strain AD169	P63165	SUMO1	9606	Homo sapiens	10684265
CPEP109	IKQEDIKP	P19893	IE2	10360	Human herpesvirus 5 strain AD169	P61956	SUMO2	9606	Homo sapiens	10684265
CPEP101	FVKSNWGLF	P16629	M	11208	Simian virus 5 (strain W3)	Q03135	CAV1	9606	Homo sapiens	20631121
CPEP110	RQARRNRRR	Q77Y21	Rev	57667	Simian-Human immunodeficiency virus	Q14974	KPNB1	9606	Homo sapiens	12614157
CPEP40	QRRGRTGRG	A1Z094	NS3	11103	Hepacivirus C	Q99873	PRMT1	9606	Homo sapiens	11483748
CPEP9	RKKRRQRRR	P04608	Tat	11706	HIV-1 M:B_HXB2R	P25963	NFKBIA	9606	Homo sapiens	22187158
CPEP9	RKKRRQRRR	P04608	Tat	11706	HIV-1 M:B_HXB2R	P55209	NAP1L1	9606	Homo sapiens	18226242
CPEP9	RKKRRQRRR	P04608	Tat	11706	HIV-1 M:B_HXB2R	P06748	NPM1	9606	Homo sapiens	9094689
CPEP27	GMFVYSPVS	Q6VGW0	E1A	28285	Human adenovirus 5	P21675	TAF1	9606	Homo sapiens	7565781
CPEP67	IQIGNHNAM	A8E1H1	RIR1	69156	Murine cytomegalovirus (strain K181)	Q9Y572	RIPK3	9606	Homo sapiens	18442983
CPEP67	IQIGNHNAM	A8E1H1	RIR1	69156	Murine cytomegalovirus (strain K181)	Q13546	RIPK1	9606	Homo sapiens	18442983
CPEP20	KELQKQITK	B9VIK5	IN	11676	Human immunodeficiency virus 1	P52292	KPNA2	9606	Homo sapiens	9275210
CPEP88	RRPSRPFRK	P13199	ICP27H	10383	Herpesvirus saimiri (strain 11)	P52292	KPNA2	9606	Homo sapiens	11278515
CPEP88	RRPSRPFRK	P13199	ICP27H	10383	Herpesvirus saimiri (strain 11)	P52292	KPNA2	9606	Homo sapiens	11278515
CPEP88	RRPSRPFRK	P13199	ICP27H	10383	Herpesvirus saimiri (strain 11)	P52294	KPNA1	9606	Homo sapiens	11278515
CPEP88	RRPSRPFRK	P13199	ICP27H	10383	Herpesvirus saimiri (strain 11)	P52294	KPNA1	9606	Homo sapiens	11278515
CPEP122	LGQGVSIEW	P69723	Vif	11706	HIV-1 M:B_HXB2R	Q9HC16	APOBEC3G	9606	Homo sapiens	20335268
CPEP122	LGQGVSIEW	P69723	Vif	11706	HIV-1 M:B_HXB2R	Q8IUX4	APOBEC3F	9606	Homo sapiens	20335268
CPEP87	PVHAGPIAP	Q71B38	Gag	11676	Human immunodeficiency virus 1	P62937	PPIA	9606	Homo sapiens	9223641
CPEP87	PVHAGPIAP	Q71B38	Gag	11676	Human immunodeficiency virus 1	P62937	PPIA	9606	Homo sapiens	8980234
CPEP23	TYGDTWAGV	Q77YF9	Vpr	11676	Human immunodeficiency virus 1	O15111	CHUK	9606	Homo sapiens	17942396
CPEP147	PEPTAPPEE	A1Z0U4	Gag	11676	Human immunodeficiency virus 1	Q99816	TSG101	9606	Homo sapiens	12379843
CPEP147	PEPTAPPEE	A1Z0U4	Gag	11676	Human immunodeficiency virus 1	Q99816	TSG101	9606	Homo sapiens	12006492
CPEP147	PEPTAPPEE	P04591	Gag	11706	HIV-1 M:B_HXB2R	Q99816	TSG101	9606	Homo sapiens	21070952
CPEP147	PEPTAPPEE	P04591	Gag	11706	HIV-1 M:B_HXB2R	Q99816	TSG101	9606	Homo sapiens	21070952
CPEP121	IVISDSEEE	F5HE22	IE2	10359	Human betaherpesvirus 5	P63165	SUMO1	9606	Homo sapiens	20519406
CPEP121	IVISDSEEE	P19893	IE2	10360	Human herpesvirus 5 strain AD169	P63165	SUMO1	9606	Homo sapiens	11264375
CPEP121	IVISDSEEE	P19893	IE2	10360	Human herpesvirus 5 strain AD169	P61956	SUMO2	9606	Homo sapiens	11264375
CPEP121	IVISDSEEE	P19893	IE2	10360	Human herpesvirus 5 strain AD169	P55854	SUMO3	9606	Homo sapiens	11264375
CPEP19	DLYCYEQLN	Q77AI1	E7	333760	Human papillomavirus type 16	P06400	RB1	9606	Homo sapiens	20088881
CPEP118	RYSKRRHGRR	Q4JQX1	IE4	341980	Human herpesvirus 3 strain Oka vaccine	Q16629	SRSF7	9606	Homo sapiens	19924249
CPEP118	RYSKRRHGRR	Q4JQX1	IE4	341980	Human herpesvirus 3 strain Oka vaccine	Q96SB4	SRPK1	9606	Homo sapiens	19924249
CPEP118	RYSKRRHGRR	Q4JQX1	IE4	341980	Human herpesvirus 3 strain Oka vaccine	Q01130	SRSF2	9606	Homo sapiens	19924249
CPEP118	RYSKRRHGRR	Q4JQX1	IE4	341980	Human herpesvirus 3 strain Oka vaccine	P84103	SRSF3	9606	Homo sapiens	19924249
CPEP133	RHIICHGGVI	Q6VGW0	E1A	28285	Human adenovirus 5	Q92793	CREBBP	9606	Homo sapiens	15741165
CPEP133	RHIICHGGVI	Q6VGW0	E1A	28285	Human adenovirus 5	Q09472	EP300	9606	Homo sapiens	15741165
CPEP116	RPEPTAPPEE	P04591	Gag	11706	HIV-1 M:B_HXB2R	Q99816	TSG101	9606	Homo sapiens	12900394
CPEP90	EVLADNLPPP	P03255	E1A	28285	Human adenovirus 5	Q14CW9	ATXN7L3	9606	Homo sapiens	26559831
CPEP90	EVLADNLPPP	P03255	E1A	28285	Human adenovirus 5	Q9UPT9	USP22	9606	Homo sapiens	26559831
CPEP90	EVLADNLPPP	P03255	E1A	28285	Human adenovirus 5	Q9NPA8	ENY2	9606	Homo sapiens	26559831
CPEP90	EVLADNLPPP	P03255	E1A	28285	Human adenovirus 5	O15265	ATXN7	9606	Homo sapiens	26559831
CPEP90	EVLADNLPPP	P03255	E1A	28285	Human adenovirus 5	Q99417	MYCBP	9606	Homo sapiens	26559831
CPEP90	EVLADNLPPP	P03255	E1A	28285	Human adenovirus 5	P01106	MYC	9606	Homo sapiens	26559831
CPEP90	EVLADNLPPP	P03255	E1A	28285	Human adenovirus 5	P61244	MAX	9606	Homo sapiens	26559831
CPEP90	EVLADNLPPP	Q6VGW0	E1A	28285	Human adenovirus 5	Q9Y4A5	TRRAP	9606	Homo sapiens	12743606
CPEP90	EVLADNLPPP	Q6VGW0	E1A	28285	Human adenovirus 5	Q96L91	EP400	9606	Homo sapiens	15741165
CPEP90	EVLADNLPPP	Q6VGW0	E1A	28285	Human adenovirus 5	Q92830	KAT2A	9606	Homo sapiens	12743606
CPEP45	EPLDLPPIIQ	D3GHM2	NS5B	11103	Hepacivirus C	Q9BW66	CINP	9606	Homo sapiens	21628470
CPEP85	ELLSGLQEWL	Q64866	E4	28284	Human adenovirus 40	P63151	PPP2R2A	9606	Homo sapiens	15775987
CPEP41	GRPPLPPKQK	Q99AU3	NS1	88776	Influenza A virus (A/Brevig Mission/1/1918	P46109	CRKL	9606	Homo sapiens	18165234
CPEP106	SEKHFRETEV	A0A1D8I0X5	Tax1	11908	Human T-cell leukemia virus type I	Q12959	DLG1	9606	Homo sapiens	18828675
CPEP106	SEKHFRETEV	P14079	Tax1	11927	Human T-cell lymphotrophic virus type 1 (	Q12959	DLG1	9606	Homo sapiens	10557085
CPEP106	SEKHFRETEV	P03409	Tax1	11926	Human T-cell lymphotrophic virus type 1 (	Q96QZ7	MAGI1	9606	Homo sapiens	26053890
CPEP106	SEKHFRETEV	P03409	Tax1	11926	Human T-cell lymphotrophic virus type 1 (	Q14160	SCRIB	9606	Homo sapiens	26053890
CPEP10	IRQGLELTLL	P19503	Env	11738	Simian immunodeficiency virus (PBJ/BC13	P63010	AP2B1	9606	Homo sapiens	9882340
CPEP129	KLVTELAENS	P0C799	P	928296	Borna disease virus-V	P09429	HMGB1	9606	Homo sapiens	14581561
CPEP139	IHTHARFLIT	P06927	E5	333760	Human papillomavirus type 16	P60903	S100A10	9606	Homo sapiens	21849434
CPEP139	IHTHARFLIT	P06927	E5	333760	Human papillomavirus type 16	O00410	IPO5	9606	Homo sapiens	18455505
CPEP139	IHTHARFLIT	P06927	E5	333760	Human papillomavirus type 16	P07355	ANXA2	9606	Homo sapiens	21849434
CPEP139	IHTHARFLIT	P06927	E5	333760	Human papillomavirus type 16	P51572	BCAP31	9606	Homo sapiens	18684816
CPEP72	SAIASGIAVS	O09720	F0	410078	Human respiratory syncytial virus S2	P61586	RHOA	9606	Homo sapiens	10438814
CPEP31	EVVLFLLNVF	P88961	vFLIP	435895	Human herpesvirus 8 type M	Q9NT62	ATG3	9606	Homo sapiens	19838173

peptide ID	peptide sequence	source accession	source name	source ncbi taxon id	source taxon	target accession	target name	target ncbi taxon id	target taxon	pmid
CPEP61	KRRKSGAKRR	P03116	E1	337052	Deltapapillomavirus 4	P52294	KPNA1	9606	Homo sapiens	17192311
CPEP61	KRRKSGAKRR	P03116	E1	337052	Deltapapillomavirus 4	O00505	KPNA3	9606	Homo sapiens	17192311
CPEP61	KRRKSGAKRR	P03116	E1	337052	Deltapapillomavirus 4	O00629	KPNA4	9606	Homo sapiens	17192311
CPEP153	GRKKRRQRRR	P04608	Tat	11706	HIV-1 M:B_HXB2R	Q09472	EP300	9606	Homo sapiens	9733868
CPEP86	SGFGPLITHG	Q786F2	H	645098	Measles virus strain Ichinose-B95a	Q13291	SLAMF1	9606	Homo sapiens	15033456
CPEP140	RKKRRQRRRA	P04608	Tat	11706	HIV-1 M:B_HXB2R	P14635	CCNB1	9606	Homo sapiens	19825974
CPEP64	CCFHCQVCFIT	P04608	Tat	11706	HIV-1 M:B_HXB2R	Q06889	EGR3	9606	Homo sapiens	11909874
CPEP64	CCFHCQVCFIT	P04608	Tat	11706	HIV-1 M:B_HXB2R	O15350	TP73	9606	Homo sapiens	16135803
CPEP13	ATIGTAMYKLL	P03209	BRLF1	10377	Human herpesvirus 4 strain B95-8	P14859	POU2F1	9606	Homo sapiens	21697476
CPEP37	FITKALGISYG	P04608	Tat	11706	HIV-1 M:B_HXB2R	Q07954	LRP1	9606	Homo sapiens	11100124
CPEP71	WKGPAKLLWKG	B9VIK5	IN	11676	Human immunodeficiency virus 1	O95373	IPO7	9606	Homo sapiens	17360709
CPEP102	PHGPVQLSYYD	P03230	LMP1	10377	Human herpesvirus 4 strain B95-8	Q15628	TRADD	9606	Homo sapiens	10409763
CPEP127	LVKPTVYVYSR	P59637	E	694009	Severe acute respiratory syndrome-relate	Q07817	BCL2L1	9606	Homo sapiens	16048439
CPEP92	QTFLHWVYCMEN	P88961	vFLIP	435895	Human herpesvirus 8 type M	Q9NT62	ATG3	9606	Homo sapiens	19838173
CPEP105	TQVDPELADQLI	P69723	Vif	11706	HIV-1 M:B_HXB2R	Q9HC16	APOBEC3G	9606	Homo sapiens	20592083
CPEP105	TQVDPELADQLI	P69723	Vif	11706	HIV-1 M:B_HXB2R	Q93034	CUL5	9606	Homo sapiens	20592083
CPEP105	TQVDPELADQLI	P69723	Vif	11706	HIV-1 M:B_HXB2R	Q8IUX4	APOBEC3F	9606	Homo sapiens	20592083
CPEP52	CFITKALGISYG	P04608	Tat	11706	HIV-1 M:B_HXB2R	Q14690	PDCD11	9606	Homo sapiens	15887232
CPEP52	CFITKALGISYG	P04608	Tat	11706	HIV-1 M:B_HXB2R	Q07954	LRP1	9606	Homo sapiens	11100124
CPEP52	CFITKALGISYG	P04608	Tat	11706	HIV-1 M:B_HXB2R	Q15020	SART3	9606	Homo sapiens	11959860
CPEP52	CFITKALGISYG	P04608	Tat	11706	HIV-1 M:B_HXB2R	Q15020	SART3	9606	Homo sapiens	11959860
CPEP3	MRHKRSAKRTKR	P03107	L2	333760	Human papillomavirus type 16	P52292	KPNA2	9606	Homo sapiens	15507604
CPEP135	KGHRGSHTMNGH	Q4F9Q5	Vif	11676	Human immunodeficiency virus 1	Q9HC16	APOBEC3G	9606	Homo sapiens	23545135
CPEP135	KGHRGSHTMNGH	P69723	Vif	11706	HIV-1 M:B_HXB2R	Q8IUX4	APOBEC3F	9606	Homo sapiens	15611076
CPEP14	ILHTPGCVPCVR	B6URY0	E1	11103	Hepacivirus C	P04114	APOB	9606	Homo sapiens	21735466
CPEP14	ILHTPGCVPCVR	B6URY0	E1	11103	Hepacivirus C	P02649	APOE	9606	Homo sapiens	21735466
CPEP28	LQLPPLERLTLD	Q77Y21	Rev	57667	Simian-Human immunodeficiency virus	P35658	NUP214	9606	Homo sapiens	10358091
CPEP28	LQLPPLERLTLD	Q77Y21	Rev	57667	Simian-Human immunodeficiency virus	O15504	NUP42	9606	Homo sapiens	10358091
CPEP28	LQLPPLERLTLD	P04618	Rev	11706	HIV-1 M:B_HXB2R	P63241	EIF5A	9606	Homo sapiens	11238447
CPEP28	LQLPPLERLTLD	P04620	Rev	11686	Human immunodeficiency virus type 1 (BF	O14980	XPO1	9606	Homo sapiens	31444273
CPEP8	RKKRRQRRRPPQ	D3GFZ4	Tat	11676	Human immunodeficiency virus 1	Q9P1T7	MDFIC	9606	Homo sapiens	16260749
CPEP142	LGIVCPICSQKP	Q77AI1	E7	333760	Human papillomavirus type 16	O15304	SIVA1	9606	Homo sapiens	17348035
CPEP74	ALGISYGRKKRRQ	P04608	Tat	11706	HIV-1 M:B_HXB2R	O60563	CCNT1	9606	Homo sapiens	11080476
CPEP74	ALGISYGRKKRRQ	P04608	Tat	11706	HIV-1 M:B_HXB2R	Q92793	CREBBP	9606	Homo sapiens	11080476
CPEP74	ALGISYGRKKRRQ	P04608	Tat	11706	HIV-1 M:B_HXB2R	Q09472	EP300	9606	Homo sapiens	11080476
CPEP63	KARHKRRNRSSRS	O92837	VP3	1891767	Macaca mulatta polyomavirus 1	P08047	SP1	9606	Homo sapiens	9466902
CPEP83	LHGLSAFSLHSYS	D3GHM2	NS5B	11103	Hepacivirus C	P12814	ACTN1	9606	Homo sapiens	14623081
CPEP95	SEWQRDQFLSQVK	P03120	E2	333760	Human papillomavirus type 16	P04637	TP53	9606	Homo sapiens	10618715
CPEP99	SWESHKSGGETRL	A3F5M8	G	11300	Rabies virus SAD B19	P29074	PTPN4	9606	Homo sapiens	22000519
CPEP99	SWESHKSGGETRL	Q08089	G	45418	Rabies virus vnukovo-32	Q6P0Q8	MAST2	9606	Homo sapiens	22894835
CPEP99	SWESHKSGGETRL	Q08089	G	45418	Rabies virus vnukovo-32	Q6P0Q8	MAST2	9606	Homo sapiens	22894835
CPEP96	GRKKRRQRRRAHQ	P04608	Tat	11706	HIV-1 M:B_HXB2R	O15111	CHUK	9606	Homo sapiens	21664225
CPEP126	TQNLYPDLSEIKK	Q66721	Gag	11665	Equine infectious anemia virus	Q8WUM4	PDCD6IP	9606	Homo sapiens	18066081
CPEP79	VLGGCRHKLVCSP	Q69027	HBx	928302	Hepatitis B virus C/ayr human/Japan/Oka	P62191	PSMC1	9606	Homo sapiens	10748218
CPEP79	VLGGCRHKLVCSP	Q69027	HBx	928302	Hepatitis B virus C/ayr human/Japan/Oka	O14818	PSMA7	9606	Homo sapiens	10748218
CPEP29	FTPPPPGYYWVPG	P0C6M3	LHDAg	261996	Hepatitis delta virus (isolate Peru-1)	Q00610	CLTC	9606	Homo sapiens	19284884
CPEP136	VTNDCPNSSIVYE	B6URY0	E1	11103	Hepacivirus C	P04114	APOB	9606	Homo sapiens	21735466
CPEP136	VTNDCPNSSIVYE	B6URY0	E1	11103	Hepacivirus C	P02649	APOE	9606	Homo sapiens	21735466
CPEP22	FPGGGQIVGGVYL	Q6UNT8	Core	11103	Hepacivirus C	O00571	DDX3X	9606	Homo sapiens	19793905
CPEP98	PLPPTKAPPIPPPR	Q913V3	NS5A	421879	Hepatitis C virus isolate HCR6	P06241	FYN	9606	Homo sapiens	14993658
CPEP98	PLPPTKAPPIPPPR	Q913V3	NS5A	421879	Hepatitis C virus isolate HCR6	P62993	GRB2	9606	Homo sapiens	14993658
CPEP98	PLPPTKAPPIPPPR	Q913V3	NS5A	421879	Hepatitis C virus isolate HCR6	P07948	LYN	9606	Homo sapiens	14993658
CPEP98	PLPPTKAPPIPPPR	Q913V3	NS5A	421879	Hepatitis C virus isolate HCR6	P06239	LCK	9606	Homo sapiens	14993658
CPEP98	PLPPTKAPPIPPPR	Q913V3	NS5A	421879	Hepatitis C virus isolate HCR6	P08631	HCK	9606	Homo sapiens	14993658
CPEP69	MRHIICHGGVITEE	Q6VGW0	E1A	28285	Human adenovirus 5	Q9Y4A5	TRRAP	9606	Homo sapiens	12743606
CPEP69	MRHIICHGGVITEE	Q6VGW0	E1A	28285	Human adenovirus 5	Q92830	KAT2A	9606	Homo sapiens	12743606
CPEP69	MRHIICHGGVITEE	Q6VGW0	E1A	28285	Human adenovirus 5	Q09472	EP300	9606	Homo sapiens	12743606
CPEP69	MRHIICHGGVITEE	Q6VGW0	E1A	28285	Human adenovirus 5	Q92831	KAT2B	9606	Homo sapiens	12743606
CPEP69	MRHIICHGGVITEE	Q6VGW0	E1A	28285	Human adenovirus 5	P06400	RB1	9606	Homo sapiens	12743606
CPEP149	EDCGTSGTQGVGSP	Q77Y21	Rev	57667	Simian-Human immunodeficiency virus	O14980	XPO1	9606	Homo sapiens	12134013
CPEP103	GCVVIVGRIVLSGK	F0UXV0	NS4A	11103	Hepacivirus C	P68104	EEF1A1	9606	Homo sapiens	16927014
CPEP89	YDVLKAACKSVIKT	Q9J5G4	vBCL2	928301	Fowlpox virus strain NVSL	Q16611	BAK1	9606	Homo sapiens	17686864
CPEP89	YDVLKAACKSVIKT	Q9J5G4	vBCL2	928301	Fowlpox virus strain NVSL	Q07812	BAX	9606	Homo sapiens	19439472

peptide ID	peptide sequence	source accession	source name	source ncbi taxon id	source taxon	target accession	target name	target ncbi taxon id	target taxon	pmid
CPEP91	MAGRSGDSDEELIR	Q77Y21	Rev	57667	Simian-Human immunodeficiency virus	P09651	HNRNPA1	9606	Homo sapiens	19808671
CPEP91	MAGRSGDSDEELIR	Q77Y21	Rev	57667	Simian-Human immunodeficiency virus	O60506	SYNCRIP	9606	Homo sapiens	19808671
CPEP94	RDVLCLRPVGAESR	Q69027	HBx	928302	Hepatitis B virus C/ayr human/Japan/Oka	O00170	AIP	9606	Homo sapiens	8972861
CPEP36	AIAEESDEEEAIVA	D2K3S2	IE1	10359	Human betaherpesvirus 5	P52630	STAT2	9606	Homo sapiens	19812155
CPEP107	GHRMAWDMMMNWSP	B6URY0	E1	11103	Hepacivirus C	P04114	APOB	9606	Homo sapiens	21735466
CPEP107	GHRMAWDMMMNWSP	B6URY0	E1	11103	Hepacivirus C	P02649	APOE	9606	Homo sapiens	21735466
CPEP51	LGNIKGILGKKDKDG	P0C6L3	SHDAg	31762	Hepatitis delta virus (ISOLATE D380)	P19338	NCL	9606	Homo sapiens	9516470
CPEP55	DLYCYEQLNDSSEEE	Q77AI1	E7	333760	Human papillomavirus type 16	P28749	RBL1	9606	Homo sapiens	8386265
CPEP55	DLYCYEQLNDSSEEE	Q77AI1	E7	333760	Human papillomavirus type 16	P06400	RB1	9606	Homo sapiens	8386265
CPEP141	LTEDRWNKPQKTKGH	P69723	Vif	11706	HIV-1 M:B_HXB2R	Q8IUX4	APOBEC3F	9606	Homo sapiens	15611076
CPEP104	EDLLNEPGQPLDLSC	P03255	E1A	28285	Human adenovirus 5	Q13363	CTBP1	9606	Homo sapiens	7479821
CPEP11	VSIEWRKKRYSTQVD	P69723	Vif	11706	HIV-1 M:B_HXB2R	Q8IUX4	APOBEC3F	9606	Homo sapiens	15611076
CPEP43	AHQNSQTHQASLSKQ	P04608	Tat	11706	HIV-1 M:B_HXB2R	P10827	THRA	9606	Homo sapiens	7609079
CPEP77	SVWIPVNEGASTSGM	F5H9D8	vIRF4	37296	Human gammaherpesvirus 8	Q93009	USP7	9606	Homo sapiens	22056774
CPEP7	SSSDSLVSPPESPVP	D2K3S2	IE1	10359	Human betaherpesvirus 5	P52630	STAT2	9606	Homo sapiens	19812155
CPEP2	MLSMFMCNNIVDYVD	P24356	F1	10254	Vaccinia virus WR	P55211	CASP9	9606	Homo sapiens	21757755
CPEP60	NKVGSLQYLALAALI	P69723	Vif	11706	HIV-1 M:B_HXB2R	Q15369	ELOC	9606	Homo sapiens	20463065
CPEP53	LERVIFPSVKIATLV	F1DT92	E4	46922	Human adenovirus 17	Q12959	DLG1	9606	Homo sapiens	9192623
CPEP46	SLQYLALAALITPKK	P69723	Vif	11706	HIV-1 M:B_HXB2R	Q15369	ELOC	9606	Homo sapiens	15574592
CPEP81	PVHAGPIAPGQMREP	P04591	Gag	11706	HIV-1 M:B_HXB2R	P62937	PPIA	9606	Homo sapiens	7969494
CPEP128	RTYGTWEDLFCDESL	B0G0V6	LT	493803	Merkel cell polyomavirus	Q96JC1	VPS39	9606	Homo sapiens	21454559
CPEP84	WGCSGKLICTTTVPW	C0KJN3	Env	11676	Human immunodeficiency virus 1	P04233	CD74	9606	Homo sapiens	22039051
CPEP42	MSKKPGGPGKNRAVN	B6V3K0	Core	11082	West Nile virus	Q9NV70	EXOC1	9606	Homo sapiens	19889084
CPEP38	QPTSQPRGDPTGPKE	P04608	Tat	11706	HIV-1 M:B_HXB2R	P29692	EEF1D	9606	Homo sapiens	9514931
CPEP65	DKELYPLTSLRSLFG	C8BSN7	Gag	11676	Human immunodeficiency virus 1	Q8WUM4	PDCD6IP	9606	Homo sapiens	18066081
CPEP65	DKELYPLTSLRSLFG	C8BSN7	Gag	11676	Human immunodeficiency virus 1	Q8WUM4	PDCD6IP	9606	Homo sapiens	14505569
CPEP25	QGPADDPGEGPSTGP	Q1HVF7	EBNA1	82830	Epstein-barr virus strain ag876	Q93009	USP7	9606	Homo sapiens	15808506
CPEP62	MNNQRKKAKNTPFNM	P14337	C	413041	Dengue virus 2 Thailand/0168/1979	Q9NV70	EXOC1	9606	Homo sapiens	19889084
CPEP66	MSSDLRLTLLELVRRL	P0DOF0	X	928296	Borna disease virus-V	P11142	HSPA8	9606	Homo sapiens	19397879
CPEP120	ISNLPYTIKYKIFEQQ	Q8QN36	CP77	265872	Cowpox virus (Brighton Red)	P63208	SKP1	9606	Homo sapiens	19211746
CPEP120	ISNLPYTIKYKIFEQQ	Q8QN36	CP77	265872	Cowpox virus (Brighton Red)	Q13616	CUL1	9606	Homo sapiens	19211746
CPEP138	PDSDPQIPPPYVEPTA	P14076	Gag	11927	Human T-cell lymphotrophic virus type 1 (	Q9H0M0	WWP1	9606	Homo sapiens	9169421
CPEP138	PDSDPQIPPPYVEPTA	P14076	Gag	11927	Human T-cell lymphotrophic virus type 1 (	O00308	WWP2	9606	Homo sapiens	9169421
CPEP112	GGLEPLSEKHFRETEV	P14079	Tax1	11927	Human T-cell lymphotrophic virus type 1 (	P78352	DLG4	9606	Homo sapiens	9482110
CPEP80	QILVESPTVLESGTKE	Q77Y21	Rev	57667	Simian-Human immunodeficiency virus	O14980	XPO1	9606	Homo sapiens	12134013
CPEP57	CMSCCRSSRTRRETQL	Q547J2	E6	333760	Human papillomavirus type 16	Q14160	SCRIB	9606	Homo sapiens	11027293
CPEP16	TATASAPPPPYVGSGL	P03327	Gag	11884	Y73 sarcoma virus	Q96QZ7	MAGI1	9606	Homo sapiens	9169421
CPEP16	TATASAPPPPYVGSGL	P03327	Gag	11884	Y73 sarcoma virus	Q9H0M0	WWP1	9606	Homo sapiens	9169421
CPEP16	TATASAPPPPYVGSGL	P03327	Gag	11884	Y73 sarcoma virus	O00308	WWP2	9606	Homo sapiens	9169421
CPEP47	ARARALARGAGPANSV	B9VQC6	ICP34.5	10298	Human alphaherpesvirus 1	P05198	EIF2S1	9606	Homo sapiens	21622569
CPEP93	TRVESENKVVILDSFD	Q14T47	NS5A	31647	Hepatitis C virus subtype 1b	P27986	PIK3R1	9606	Homo sapiens	14709551
CPEP1	YRLRFSKRDARRERIR	Q4JQX1	IE4	341980	Human herpesvirus 3 strain Oka vaccine	Q16629	SRSF7	9606	Homo sapiens	19924249
CPEP1	YRLRFSKRDARRERIR	Q4JQX1	IE4	341980	Human herpesvirus 3 strain Oka vaccine	Q01130	SRSF2	9606	Homo sapiens	19924249
CPEP1	YRLRFSKRDARRERIR	Q4JQX1	IE4	341980	Human herpesvirus 3 strain Oka vaccine	P84103	SRSF3	9606	Homo sapiens	19924249
CPEP6	RKVKKKIKKLEDEHPW	P0C6L3	SHDAg	31762	Hepatitis delta virus (ISOLATE D380)	P19338	NCL	9606	Homo sapiens	9516470
CPEP124	SAPCLLKLSTTLLCLEI	O41960	M8	33708	Murid gammaherpesvirus 4	P21796	VDAC1	9606	Homo sapiens	18069888
CPEP15	EIRLKVFVLGGCRHKLV	Q69027	HBx	928302	Hepatitis B virus C/ayr human/Japan/Oka	P25054	APC	9606	Homo sapiens	20971552
CPEP125	DLYCYEQLNDSSEEEDE	Q77AI1	E7	333760	Human papillomavirus type 16	P68400	CSNK2A1	9606	Homo sapiens	2153075
CPEP125	DLYCYEQLNDSSEEEDE	Q77AI1	E7	333760	Human papillomavirus type 16	P06400	RB1	9606	Homo sapiens	2153075
CPEP5	MGSSGTRQVTQASSFTW	F5H9D8	vIRF4	37296	Human gammaherpesvirus 8	Q93009	USP7	9606	Homo sapiens	22056774
CPEP5	MGSSGTRQVTQASSFTW	F5H9D8	vIRF4	37296	Human gammaherpesvirus 8	Q93009	USP7	9606	Homo sapiens	22056774
CPEP137	GPSDSGAGRAAEDRKCL	D3YPC5	ICP22	10298	Human alphaherpesvirus 1	Q9NZM5	NOP53	9606	Homo sapiens	10196275
CPEP73	EALKKALRRHRFLWQRR	F5HI84	UL37	10359	Human betaherpesvirus 5	Q07812	BAX	9606	Homo sapiens	15004026
CPEP100	ACTNCYCKKCCFHCQVC	P04608	Tat	11706	HIV-1 M:B_HXB2R	P28799	GRN	9606	Homo sapiens	10079180
CPEP100	ACTNCYCKKCCFHCQVC	P04608	Tat	11706	HIV-1 M:B_HXB2R	P28799	GRN	9606	Homo sapiens	10079180
CPEP114	DILDLWIYHTQGYFPDWQ	P04601	Nef	11706	HIV-1 M:B_HXB2R	O14734	ACOT8	9606	Homo sapiens	10799608
CPEP115	HFEPPTLHELYDLDVTAP	P03255	E1A	28285	Human adenovirus 5	P06400	RB1	9606	Homo sapiens	1534854
CPEP115	HFEPPTLHELYDLDVTAP	E1U5L3	E1A	10534	Human adenovirus 6	P28749	RBL1	9606	Homo sapiens	1331501
CPEP115	HFEPPTLHELYDLDVTAP	E1U5L3	E1A	10534	Human adenovirus 6	P78396	CCNA1	9606	Homo sapiens	1331501
CPEP115	HFEPPTLHELYDLDVTAP	E1U5L3	E1A	10534	Human adenovirus 6	P06400	RB1	9606	Homo sapiens	1331501
CPEP115	HFEPPTLHELYDLDVTAP	E1U5L3	E1A	10534	Human adenovirus 6	Q08999	RBL2	9606	Homo sapiens	1331501
CPEP39	VHIPLGDARLVITTYWGL	P69723	Vif	11706	HIV-1 M:B_HXB2R	Q9HC16	APOBEC3G	9606	Homo sapiens	18619467

Table S2. Pathways significantly enriched in the list of proteins targeted by the viral peptide library. Pathways were selected with a corrected p-value above 0.05 and clustered using the DAVID web server(59).

**Cluster 1**	
**INTRACELLULAR TRANSPORT**	
GOTERM_CC_DIRECT	GO:0005643~nuclear pore
GOTERM_BP_DIRECT	GO:0075733~intracellular transport of virus
GOTERM_MF_DIRECT	GO:0008139~nuclear localization sequence binding
GOTERM_BP_DIRECT	GO:0006607~NLS-bearing protein import into nucleus
GOTERM_MF_DIRECT	GO:0008565~protein transporter activity
GOTERM_BP_DIRECT	GO:0006406~mRNA export from nucleus
**Cluster 2**	
**APOPTOSIS**	
GOTERM_BP_DIRECT	GO:0034644~cellular response to UV
GOTERM_BP_DIRECT	GO:0008630~intrinsic apoptotic signaling pathway in response to DNA damage
GOTERM_BP_DIRECT	GO:0010332~response to gamma radiation
KEGG_PATHWAY	hsa04210:Apoptosis
GOTERM_BP_DIRECT	GO:1901796~regulation of signal transduction by p53 class mediator
GOTERM_BP_DIRECT	GO:0097296~activation of cysteine-type endopeptidase activity involved in apoptotic signaling pathway
GOTERM_BP_DIRECT	GO:0006919~activation of cysteine-type endopeptidase activity involved in apoptotic process
GOTERM_BP_DIRECT	GO:0097191~extrinsic apoptotic signaling pathway
GOTERM_BP_DIRECT	GO:0071550~death-inducing signaling complex assembly
GOTERM_BP_DIRECT	GO:0008630~intrinsic apoptotic signaling pathway in response to DNA damage
**Cluster 3**	
**VIRUS**	
KEGG_PATHWAY	hsa05169:Epstein-Barr virus infection
KEGG_PATHWAY	hsa05160:Hepatitis C
GOTERM_BP_DIRECT	GO:0019054~modulation by virus of host process
**Cluster 4**	
**INNATE IMMUNITY**	
KEGG_PATHWAY	hsa04064:NF-kappa B signaling pathway
KEGG_PATHWAY	hsa04668:TNF signaling pathway
KEGG_PATHWAY	hsa04622:RIG-I-like receptor signaling pathway
KEGG_PATHWAY	hsa04622:RIG-I-like receptor signaling pathway
**Cluster 5**	
**CANCER**	
KEGG_PATHWAY	hsa05222:Small cell lung cancer
KEGG_PATHWAY	hsa05211:Renal cell carcinoma
KEGG_PATHWAY	hsa05200:Pathways in cancer

KEGG_PATHWAY	hsa05220:Chronic myeloid leukemia
KEGG_PATHWAY	hsa05222:Small cell lung cancer
KEGG_PATHWAY	hsa05215:Prostate cancer
KEGG_PATHWAY	hsa05210:Colorectal cancer
KEGG_PATHWAY	hsa05212:Pancreatic cancer
KEGG_PATHWAY	hsa05213:Endometrial cancer
KEGG_PATHWAY	hsa05211:Renal cell carcinoma
GOTERM_BP_DIRECT	GO:0050900~leukocyte migration

Cluster 5

PATHWAYS

GOTERM_BP_DIRECT	GO:0050852~T cell receptor signaling pathway
GOTERM_BP_DIRECT	GO:0038095~Fc-epsilon receptor signaling pathway
KEGG_PATHWAY	hsa04660:T cell receptor signaling pathway
GOTERM_BP_DIRECT	GO:0002223~stimulatory C-type lectin receptor signaling pathway
KEGG_PATHWAY	hsa04919:Thyroid hormone signaling pathway
KEGG_PATHWAY	hsa04068:FoxO signaling pathway

Cluster 6

MISCELLANEOUS

GOTERM_CC_DIRECT	GO:0031234~extrinsic component of cytoplasmic side of plasma membrane
GOTERM_BP_DIRECT	GO:0038083~peptidyl-tyrosine autophosphorylation
GOTERM_MF_DIRECT	GO:0004715~non-membrane spanning protein tyrosine kinase activity
GOTERM_CC_DIRECT	GO:0005913~cell-cell adherens junction
KEGG_PATHWAY	hsa04380:Osteoclast differentiation

Table S3. Activities of the viral peptide library in a dose-response experiment against influenza A virus replication in A549 cells. 48 hr post-treatment the neuraminidase activity was quantified in the supernatant. Values are normalized to those of cells treated by vehicle.

µM	0,823	2,4691	7,4074	22,2	66,6667	200
CPEP131	1,0236	1,0486	0,9867	1,0471	1,1075	1,0859
CPEP130	0,8445	0,8513	0,8415	0,933	1,0245	1,0055
CPEP152	0,8889	0,8832	0,8045	0,9121	1,0197	1,0605
CPEP21	0,8221	0,8753	0,7869	0,8963	1,0057	0,9829
CPEP44	0,8116	0,843	0,7952	0,9052	1,0152	0,8825
CPEP33	0,7063	0,8619	0,7735	0,8914	1,0094	0,999
CPEP132	0,8549	0,87	0,8306	0,9192	1,0078	0,8244
CPEP150	0,912	0,7746	0,8703	0,9305	0,9907	1,0149
CPEP48	0,9611	0,8941	0,9555	1,0228	1,0901	1,01
CPEP154	1,0316	0,9855	1,0107	1,0253	1,0399	1,0928
CPEP54	1,0199	1,1627	0,6425	0,844	1,0456	1,1329
**µM**	**0,823**	**2,4691**	**7,4074**	**22,2**	**66,6667**	**200**
CPEP70	0,9822	0,9261	0,9473	0,9863	1,0254	1,0392
CPEP12	0,7557	0,7112	0,7657	0,8312	0,8968	0,732
CPEP123	0,7776	0,7299	0,7535	0,8404	0,9273	0,78
CPEP49	0,6686	0,6943	0,7716	0,8389	0,9061	0,7922
CPEP143	0,6928	0,7636	0,8039	0,8423	0,8807	0,8484
CPEP58	0,7664	0,7115	0,806	0,8252	0,8444	0,7155
CPEP97	0,83	0,6842	0,8009	0,836	0,8711	0,8478
CPEP30	0,957	0,8714	0,8974	0,923	0,9486	0,9647
CPEP59	1,1044	1,0801	1,0995	1,0974	1,0953	1,1965
CPEP113	0,965	0,9338	0,9116	0,979	1,0465	0,9723
CPEP17	0,7778	0,6648	0,7257	0,813	0,9002	0,7956
**µM**	**0,823**	**2,4691**	**7,4074**	**22,2**	**66,6667**	**200**
CPEP35	0,8082	0,6924	0,7544	0,7983	0,8423	0,7387
CPEP68	0,7462	0,7296	0,7652	0,8576	0,95	0,7574
CPEP26	0,7392	0,7297	0,8073	0,8236	0,8398	0,7568
CPEP18	0,8201	0,7017	0,7944	0,7825	0,7706	0,7098
CPEP119	0,8876	0,7125	0,8133	0,8371	0,8609	0,8047
CPEP34	0,9473	0,8162	0,8714	0,8779	0,8844	0,8588
CPEP56	1,0162	0,8416	0,8936	0,9231	0,9527	0,9628
CPEP32	1,0838	1,0337	1,1078	1,0576	1,0074	1,0785
CPEP151	0,9906	0,9123	0,9111	0,9273	0,9435	1,016
CPEP82	0,801	0,6968	0,5924	0,7544	0,9164	0,8479
CPEP146	0,8163	0,7842	0,7775	0,8498	0,9221	0,7481
**µM**	**0,823**	**2,4691**	**7,4074**	**22,2**	**66,6667**	**200**
CPEP117	0,8345	0,8234	0,8773	0,8782	0,8792	0,72
CPEP76	0,7594	0,7722	0,7943	0,787	0,7797	0,7678
CPEP148	0,8071	0,7477	0,8788	0,8272	0,7757	0,7433
CPEP75	0,8942	0,8135	0,8547	0,8062	0,7577	0,6614
CPEP50	0,883	0,771	0,9007	0,8095	0,7183	0,2471
CPEP24	0,8674	0,743	0,789	0,8114	0,8339	0,7408
CPEP111	0,9384	0,8026	0,8887	0,8898	0,8909	0,7987
CPEP78	0,9859	0,8283	0,8644	0,8841	0,9037	0,663
CPEP145	0,8361	0,7253	0,7634	0,839	0,9146	0,7911
CPEP144	1,0036	0,8026	0,9626	0,9081	0,8536	0,7318
CPEP108	0,7112	0,8479	0,9625	0,9027	0,8429	0,7289
**µM**	**0,823**	**2,4691**	**7,4074**	**22,2**	**66,6667**	**200**
CPEP4	0,9249	0,8509	0,9749	0,8431	0,7112	0,6513
CPEP134	0,9416	0,8887	0,9196	0,8116	0,7036	0,426
CPEP109	0,9055	0,8881	0,975	0,889	0,8029	0,7052
CPEP101	1,0136	0,9061	0,9057	0,9003	0,8948	0,7463
CPEP110	1,0272	0,9228	0,9411	0,8952	0,8493	0,8723
CPEP40	1,0647	0,9944	1,0761	1,004	0,9318	0,9281
CPEP9	1,06	0,9262	0,9107	0,9823	1,0539	1,0716
CPEP27	0,9653	0,7997	0,7763	0,8123	0,8484	0,2565
CPEP67	0,9632	0,8279	0,768	0,5849	0,4017	0,0404
CPEP20	0,9855	0,8291	0,8347	0,8116	0,7886	0,6411
CPEP88	0,9984	0,8217	0,8463	0,7716	0,6969	0,55

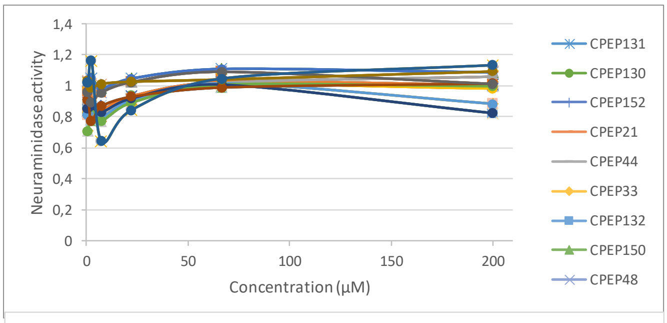

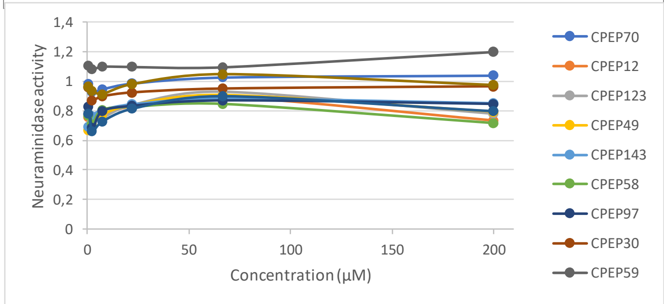

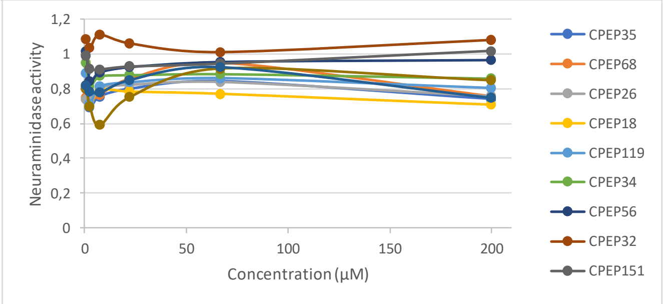

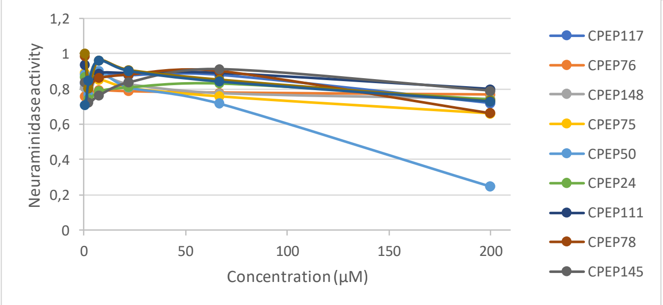

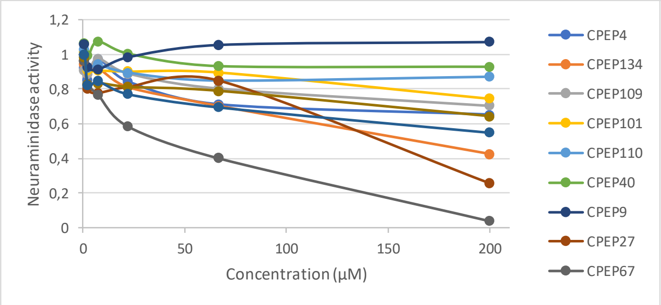

**µM**	**0,823**	**2,4691**	**7,4074**	**22,2**	**66,6667**	**200**
**CPEP122**	1,0256	0,9073	0,9191	0,854	0,7889	0,6921
**CPEP87**	1,0155	0,8845	0,906	0,8043	0,7025	0,6355
**CPEP23**	1,0478	0,9197	0,9274	0,8391	0,7507	0,5681
**CPEP147**	1,0079	0,8793	0,9284	0,8248	0,7212	0,6487
**CPEP121**	1,0507	1,0097	0,9639	1,0096	1,0552	1,0697
**CPEP19**	0,9731	0,9188	0,831	0,81	0,789	0,1727
**CPEP118**	0,9639	0,9014	0,7974	0,7875	0,7776	0,7942
**CPEP133**	0,951	0,9186	0,811	0,4954	0,1797	0,0947
**CPEP116**	0,9564	0,8677	0,7015	0,683	0,6644	0,7624
**CPEP90**	0,9901	0,9469	0,8328	0,7822	0,7316	0,7518
**CPEP45**	0,9966	0,9443	0,9188	0,8381	0,7574	0,7336
**µM**	**0,823**	**2,4691**	**7,4074**	**22,2**	**66,6667**	**200**
**CPEP85**	0,9858	0,9371	0,8683	0,8356	0,8029	0,9118
**CPEP41**	1,0727	0,9209	0,9892	0,912	0,8347	0,8561
**CPEP106**	0,9396	0,9387	1,0552	1,0803	1,1055	1,1108
**CPEP10**	1,1254	1,1442	0,9853	0,9579	0,9305	0,8494
**CPEP129**	1,1002	1,0838	0,9983	0,8882	0,7782	0,8014
**CPEP139**	1,1262	1,0704	1,0144	0,9135	0,8125	0,8832
**CPEP72**	1,0538	1,1217	1,1419	1,1913	1,1769	1,0607
**CPEP31**	0,7352	0,951	0,7335	0,2851	0,114	0,0494
**CPEP61**	1,0166	1,0813	0,9769	1,0577	1,1022	0,867
**CPEP153**	1,0171	1,0648	1,0014	1,0832	1,1029	0,9878
**CPEP86**	0,7964	1,0728	0,943	1,0588	1,0345	0,9563
**µM**	**0,823**	**2,4691**	**7,4074**	**22,2**	**66,6667**	**200**
**CPEP140**	0,9961	1,0685	0,9007	1,0982	1,0124	0,616
**CPEP64**	0,8369	0,9654	0,5226	0,1905	0,0909	0,0708
**CPEP13**	0,7537	1,0623	0,9134	1,0033	0,9233	0,2151
**CPEP37**	0,8663	1,1047	1,0912	1,1287	1,1558	0,8898
**CPEP71**	0,7606	1,0227	0,9266	1,0288	0,9918	0,1137
**CPEP102**	0,6759	1,0283	0,877	0,942	1,0125	0,8099
**CPEP127**	0,9001	1,007	0,8577	0,8705	0,9535	0,2557
**CPEP92**	0,9306	1,0232	0,9375	0,7005	0,3098	0,0592
**CPEP105**	0,8996	1,0337	0,8652	0,8935	0,7563	1,0274
**CPEP52**	0,9576	1,1215	0,9392	1,0146	0,5319	0,1673
**CPEP3**	0,9751	1,0464	1,0155	1,1006	1,1381	1,0134
**µM**	**0,823**	**2,4691**	**7,4074**	**22,2**	**66,6667**	**200**
**CPEP135**	0,9322	0,9359	0,8333	0,9598	0,9822	0,865
**CPEP14**	0,9272	0,9392	0,9017	1,0033	1,0554	0,9254
**CPEP28**	0,6533	0,9639	0,9303	0,9895	1,0323	0,819
**CPEP8**	0,9586	1,0378	0,9697	0,9778	1,0084	0,8198
**CPEP142**	0,9385	0,9538	0,9445	0,9689	1,085	0,8923
**CPEP74**	0,913	1,0192	0,9165	0,9469	0,9895	0,8907
**CPEP63**	0,6161	1	0,9177	0,9172	0,9488	0,8323
**CPEP83**	0,6779	0,9223	0,934	0,9424	0,7894	0,249
**CPEP95**	0,9256	0,9776	0,9237	0,9538	1,0019	0,9927
**CPEP99**	0,9063	0,994	0,9109	1,004	1,1024	1,0243
**CPEP96**	0,9089	0,9539	0,8942	0,9748	0,9344	0,8043
**µM**	**0,823**	**2,4691**	**7,4074**	**22,2**	**66,6667**	**200**
**CPEP126**	0,9498	0,9431	0,8627	0,8904	0,9364	0,8408
**CPEP79**	0,8781	0,9501	0,8283	0,8549	0,8194	0,8351
**CPEP29**	0,8366	0,937	0,8169	0,9093	0,9126	0,8546
**CPEP136**	0,8343	0,9183	0,8168	0,8466	0,9818	0,9602
**CPEP22**	1,123	1,157	1,0052	0,904	0,8029	0,7797
**CPEP98**	0,8951	0,9959	0,9005	0,9384	0,9857	1,0266
**CPEP69**	0,9574	1,0075	1,0635	1,0597	1,1613	1,1006
**CPEP149**	0,9141	0,9731	0,9544	0,9641	0,9908	0,9593
**CPEP103**	0,8342	0,8866	0,7276	0,2828	0,1254	0,0978
**CPEP89**	0,8515	0,8767	0,7891	0,6685	0,2057	0,097
**CPEP91**	0,8065	0,8956	0,8592	0,8658	0,8689	0,8049

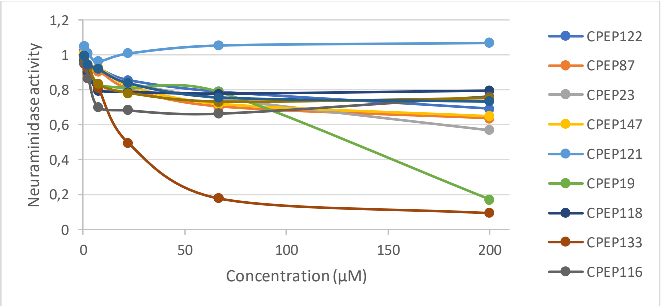

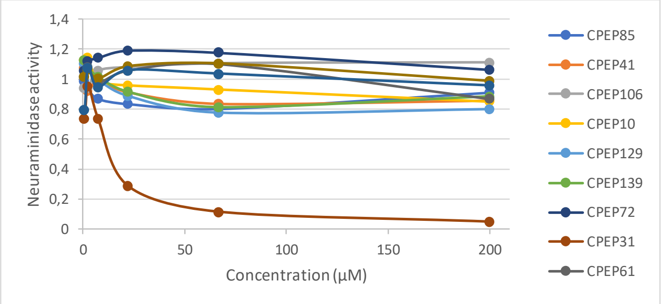

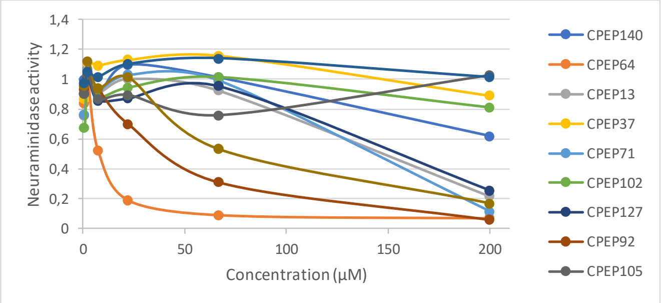

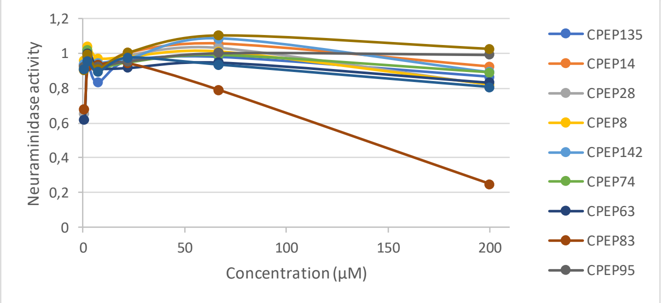

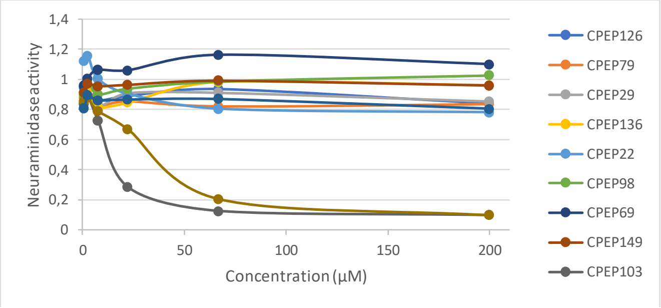

µM	0,823	2,4691	7,4074	22,2	66,6667	200
**CPEP94**	0,7797	0,8827	0,8423	0,8926	0,695	0,3954
**CPEP36**	0,779	0,9289	0,7742	0,8636	0,6845	0,6163
**CPEP107**	0,7424	0,9047	0,8224	0,9099	0,2891	0,108
**CPEP51**	0,8902	0,9673	0,9421	0,9184	1,0486	1,0387
**CPEP55**	0,8699	0,9062	0,9392	0,9333	1,0033	1,025
**CPEP141**	0,8505	0,92	0,9289	0,9378	0,9241	0,9005
**CPEP104**	0,8247	0,9133	0,8771	0,8954	0,8952	0,8372
**CPEP11**	0,82	0,9458	0,8342	0,918	0,791	0,401
**CPEP43**	0,7858	0,876	0,8511	0,9843	0,7841	0,8034
**CPEP77**	0,7817	0,8942	0,7709	0,9377	0,8131	0,8793
**CPEP7**	0,7414	0,8401	0,8042	0,9561	0,728	0,9039
**µM**	**0,823**	**2,4691**	**7,4074**	**22,2**	**66,6667**	**200**
**CPEP2**	0,7958	0,7977	0,455	0,3325	0,1212	0,0867
**CPEP60**	0,8991	1,0157	0,7801	0,7636	0,247	0,117
**CPEP53**	0,9211	1,008	1,0347	1,0363	1,0977	0,9303
**CPEP46**	0,9309	1,043	1,0188	0,9118	0,8482	0,1529
**CPEP81**	0,9268	1,0385	1,0221	1,0319	1,026	0,9395
**CPEP128**	0,9138	1,0131	0,9772	1,0305	1,0247	0,6933
**CPEP84**	0,9093	0,9765	0,9455	0,9299	0,3888	0,0819
**CPEP42**	0,9146	0,9945	0,9864	1,055	1,0215	0,9312
**CPEP38**	0,8756	0,9701	0,8933	1,0277	0,9904	0,7596
**CPEP65**	0,8623	0,9706	0,9802	1,0442	1,0044	0,356
**CPEP25**	0,8914	0,9402	0,9062	1,0318	1,0076	0,8837
**µM**	**0,823**	**2,4691**	**7,4074**	**22,2**	**66,6667**	**200**
**CPEP62**	1,1108	1,0767	0,9136	0,891	1,1387	1,2311
**CPEP66**	1,0081	1,0026	1,0851	1,2015	1,0904	1,1231
**CPEP120**	1,0562	0,9901	1,0754	1,193	1,0268	1,0456
**CPEP138**	1,063	0,9934	1,0804	1,1918	1,0379	1,0087
**CPEP112**	1,0673	1,0388	1,1047	1,1545	1,0264	1,0677
**CPEP80**	1,0444	1,0137	1,0974	1,1492	0,9708	0,8685
**CPEP57**	1,0672	0,702	1,111	1,1821	0,9599	0,9828
**CPEP16**	1,1052	1,0207	1,1179	1,2026	1,0841	1,0804
**CPEP47**	1,0745	1,0399	1,1089	1,1923	1,0424	1,0179
**CPEP93**	1,1071	1,0829	1,1322	1,1929	1,0883	1,1147
**CPEP1**	1,1315	1,0715	1,1426	1,1631	1,1178	1,1583
**µM**	**0,823**	**2,4691**	**7,4074**	**22,2**	**66,6667**	**200**
**CPEP6**	1,0694	0,9677	1,125	1,0958	1,0797	1,0809
**CPEP124**	1,0611	0,9657	1,0354	0,6181	0,1507	0,1059
**CPEP15**	1,1075	0,9773	1,0815	1,094	0,4276	0,1561
**CPEP125**	1,0772	0,9577	1,1015	1,1403	0,8979	0,7813
**CPEP5**	1,086	0,9863	1,1252	1,1837	1,0696	1,0247
**CPEP137**	1,0904	0,9326	1,1347	1,189	1,0155	1,0777
**CPEP73**	1,11	0,9956	1,1329	1,1241	0,4622	0,1338
**CPEP100**	1,092	0,9938	1,0674	1,1329	1,0274	1,0893
**CPEP114**	1,1783	1,0928	1,195	1,017	0,5539	0,4016
**CPEP115**	1,141	1,0495	1,1676	1,1784	1,1762	1,0337
**CPEP39**	1,1661	1,1357	1,0416	0,3163	0,0606	0,0415

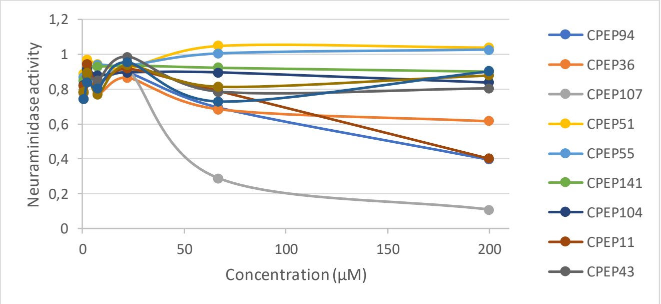

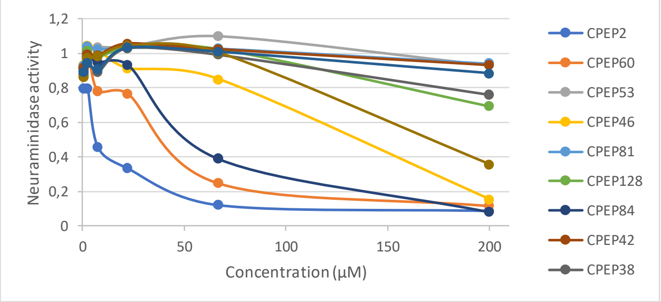

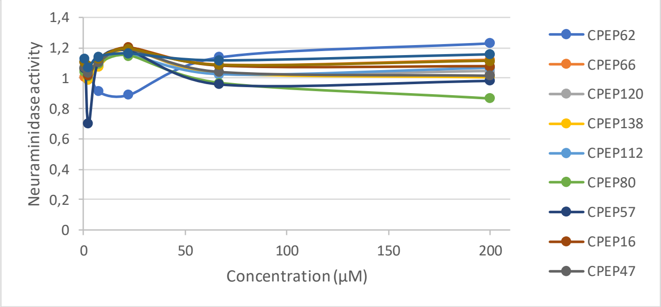

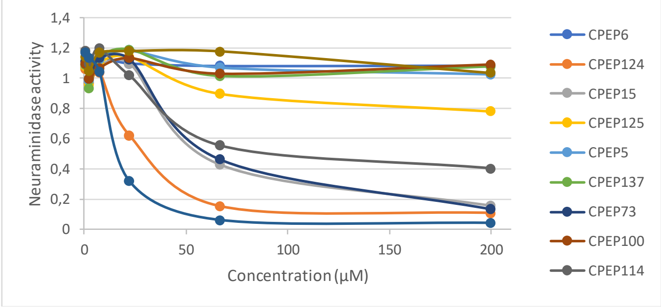

Dataset Legends

Dataset S1. Matrix of Gene Ontology biological processes versus viruses having more than 150 vh PPI. 1 means the list of human proteins interacting with the virus is significantly enriched for proteins involved in the biological process, 0 means it is not. Pathways were retrieved using GOATOOLS python library(58), automating enrichment analyses of multiple lists of human proteins. The Gene Ontology set used was GO Biological Processes (1st sheet) or GOslim Biological Processes (2nd sheet). Processes were filtered out using a 0.05 threshold on Benjamini Hochberg corrected p-values.

Dataset S2. Viral peptides containing a SLiM as described in the Eukaryotic Linear Motif Resource(21). Peptides are described with their sequence, their viral source(s) and their human target(s). SLiMs are described with their accession, their functional site name and their regular expression.

Dataset S3. All peptide activities in different screens. Each peptide is described with its sequence, its source (protein accession name and taxon), its target(s) (protein accession and name) as well as the PMID of the publication where it has been originally described as interacting with the target. Activities values are normalized to those of cells treated with vehicle.

SI References

1. M. Zahn-Zabal, et al., The neXtProt knowledgebase in 2020: data, tools and usability improvements. Nucleic Acids Res 48, D328–D334 (2020).
2. H. Hermjakob, et al., The HUPO PSI's molecular interaction format--a community standard for the representation of protein interaction data. Nat Biotechnol 22, 177–83 (2004).
3. E. Gasteiger, E. Jung, A. Bairoch, SWISS-PROT: connecting biomolecular knowledge via a protein database. Curr Issues Mol Biol 3, 47–55 (2001).
4. UniProt Consortium, UniProt: the Universal Protein Knowledgebase in 2023. Nucleic Acids Res (2022) https:/doi.org/10.1093/nar/gkac1052.
5. H. Hermjakob, et al., IntAct: an open source molecular interaction database. Nucleic Acids Res 32, D452-5 (2004).
6. T. Guirimand, S. Delmotte, V. Navratil, VirHostNet 2.0: surfing on the web of virus/host molecular interactions data. Nucleic Acids Res 43, D583-7 (2015).
7. X. Yang, et al., HVIDB: a comprehensive database for human-virus protein-protein interactions. Brief Bioinform 22, 832–844 (2021).
8. N. Stern-Ginossar, S. R. Thompson, M. B. Mathews, I. Mohr, Translational Control in Virus-Infected Cells. Cold Spring Harb Perspect Biol 11 (2019).
9. M. E. Nemeroff, S. M. Barabino, Y. Li, W. Keller, R. M. Krug, Influenza virus NS1 protein interacts with the cellular 30 kDa subunit of CPSF and inhibits 3'end formation of cellular pre-mRNAs. Mol Cell 1, 991–1000 (1998).
10. N. J. Moorman, et al., Human cytomegalovirus protein UL38 inhibits host cell stress responses by antagonizing the tuberous sclerosis protein complex. Cell Host Microbe 3, 253–62 (2008).
11. C. McKinney, et al., Global reprogramming of the cellular translational landscape facilitates cytomegalovirus replication. Cell Rep 6, 9–17 (2014).
12. A. Donchet, E. Vassal-Stermann, F. C. A. Gérard, R. W. H. Ruigrok, T. Crépin, Differential Behaviours and Preferential Bindings of Influenza Nucleoproteins on Importins-α. Viruses 12 (2020).
13. D. J. Goodwin, A. Whitehouse, A gamma-2 herpesvirus nucleocytoplasmic shuttle protein interacts with importin alpha 1 and alpha 5. J Biol Chem 276, 19905–12 (2001).
14. C. Wu, et al., Systematic identification of SH3 domain-mediated human protein-protein interactions by peptide array target screening. Proteomics 7, 1775–85 (2007).
15. S. A. Cunningham, M. N. Waxham, P. M. Arrate, T. A. Brock, Interaction of the Flt-1 tyrosine kinase receptor with the p85 subunit of phosphatidylinositol 3-kinase. Mapping of a novel site involved in binding. J Biol Chem 270, 20254–7 (1995).

16. K. A. Wong, et al., Intersectin (ITSN) family of scaffolds function as molecular hubs in protein interaction networks. PLoS One 7, e36023 (2012).
17. A. Street, A. Macdonald, K. Crowder, M. Harris, The Hepatitis C virus NS5A protein activates a phosphoinositide 3-kinase-dependent survival signaling cascade. J Biol Chem 279, 12232–41 (2004).
18. H. Korkaya, et al., The ORF3 protein of hepatitis E virus binds to Src homology 3 domains and activates MAPK. J Biol Chem 276, 42389–400 (2001).
19. M. Pires de Miranda, F. B. Lopes, C. E. McVey, X. R. Bustelo, J. P. Simas, Role of Src homology domain binding in signaling complexes assembled by the murid γ-herpesvirus M2 protein. J Biol Chem 288, 3858–70 (2013).
20. T. Linnemann, Y.-H. Zheng, R. Mandic, B. M. Peterlin, Interaction between Nef and phosphatidylinositol-3-kinase leads to activation of p21-activated kinase and increased production of HIV. Virology 294, 246–55 (2002).
21. M. Kumar, et al., ELM-the eukaryotic linear motif resource in 2020. Nucleic Acids Res 48, D296–D306 (2020).
22. A. D. Rouillard, et al., The harmonizome: a collection of processed datasets gathered to serve and mine knowledge about genes and proteins. Database (Oxford) 2016 (2016).
23. Y. Ivarsson, et al., Large-scale interaction profiling of PDZ domains through proteomic peptide-phage display using human and viral phage peptidomes. Proc Natl Acad Sci U S A 111, 2542–7 (2014).
24. M. Sheng, C. Sala, PDZ domains and the organization of supramolecular complexes. Annu Rev Neurosci 24, 1–29 (2001).
25. R. Lin, et al., HHV-8 encoded vIRF-1 represses the interferon antiviral response by blocking IRF-3 recruitment of the CBP/p300 coactivators. Oncogene 20, 800–11 (2001).
26. X. Yu, et al., VHL negatively regulates SARS coronavirus replication by modulating nsp16 ubiquitination and stability. Biochem Biophys Res Commun 459, 270–276 (2015).
27. P. Massimi, D. Pim, C. Bertoli, V. Bouvard, L. Banks, Interaction between the HPV-16 E2 transcriptional activator and p53. Oncogene 18, 7748–54 (1999).
28. R. J. Kraus, et al., Reactivation of Epstein-Barr Virus by HIF-1α Requires p53. J Virol 94 (2020).
29. O. Pornillos, et al., HIV Gag mimics the Tsg101-recruiting activity of the human Hrs protein. J Cell Biol 162, 425–34 (2003).
30. H.-H. Chua, et al., Role of the TSG101 gene in Epstein-Barr virus late gene transcription. J Virol 81, 2459–71 (2007).
31. I. Ote, et al., Varicella-zoster virus IE4 protein interacts with SR proteins and exports mRNAs through the TAP/NXF1 pathway. PLoS One 4, e7882 (2009).

32. Z. Ao, et al., Interaction of human immunodeficiency virus type 1 integrase with cellular nuclear import receptor importin 7 and its impact on viral replication. J Biol Chem 282, 13456–67 (2007).
33. F. Juillard, et al., Epstein-Barr virus protein EB2 stimulates cytoplasmic mRNA accumulation by counteracting the deleterious effects of SRp20 on viral mRNAs. Nucleic Acids Res 40, 6834–49 (2012).
34. Y.-C. Yang, B. Sugden, Epstein-Barr Virus Limits the Accumulation of IPO7, an Essential Gene Product. Front Microbiol 12, 643327 (2021).
35. H.-R. Lee, et al., Bilateral inhibition of HAUSP deubiquitinase by a viral interferon regulatory factor protein. Nat Struct Mol Biol 18, 1336–44 (2011).
36. A. Ali, R. Raja, S. R. Farooqui, S. Ahmad, A. C. Banerjea, USP7 deubiquitinase controls HIV-1 production by stabilizing Tat protein. Biochem J 474, 1653–1668 (2017).
37. Y.-H. Kou, et al., Hepatitis C virus NS4A inhibits cap-dependent and the viral IRES-mediated translation through interacting with eukaryotic elongation factor 1A. J Biomed Sci 13, 861–74 (2006).
38. D. Li, et al., Specific Interaction between eEF1A and HIV RT Is Critical for HIV-1 Reverse Transcription and a Potential Anti-HIV Target. PLoS Pathog 11, e1005289 (2015).
39. M. K. Pastey, J. E. Crowe, B. S. Graham, RhoA interacts with the fusion glycoprotein of respiratory syncytial virus and facilitates virus-induced syncytium formation. J Virol 73, 7262–70 (1999).
40. L. Wang, et al., Modulation of HIV-1 replication by a novel RhoA effector activity. J Immunol 164, 5369–74 (2000).
41. E. Yu, et al., Structural determinants of caspase-9 inhibition by the vaccinia virus protein, F1L. J Biol Chem 286, 30748–30758 (2011).
42. A. M. Chinnaiyan, C. Woffendin, V. M. Dixit, G. J. Nabel, The inhibition of pro-apoptotic ICE-like proteases enhances HIV replication. Nat Med 3, 333–7 (1997).
43. P. Mannová, et al., Modification of host lipid raft proteome upon hepatitis C virus replication. Mol Cell Proteomics 5, 2319–25 (2006).
44. Y.-J. Song, K. M. Izumi, N. P. Shinners, B. E. Gewurz, E. Kieff, IRF7 activation by Epstein-Barr virus latent membrane protein 1 requires localization at activation sites and TRAF6, but not TRAF2 or TRAF3. Proc Natl Acad Sci U S A 105, 18448–53 (2008).
45. T. Zhang, et al., Hepatitis C virus inhibits intracellular interferon alpha expression in human hepatic cell lines. Hepatology 42, 819–27 (2005).
46. M. S. Darshan, J. Lucchi, E. Harding, J. Moroianu, The l2 minor capsid protein of human papillomavirus type 16 interacts with a network of nuclear import receptors. J Virol 78, 12179–88 (2004).

47. C. J. Neufeldt, et al., Hepatitis C virus-induced cytoplasmic organelles use the nuclear transport machinery to establish an environment conducive to virus replication. PLoS Pathog 9, e1003744 (2013).
48. P. Wallet, et al., IFN-γ extends the immune functions of Guanylate Binding Proteins to inflammasome-independent antibacterial activities during Francisella novicida infection. PLoS Pathog 13, e1006630 (2017).
49. M. Ozanic, V. Marecic, Y. Abu Kwaik, M. Santic, The Divergent Intracellular Lifestyle of Francisella tularensis in Evolutionarily Distinct Host Cells. PLoS Pathog 11, e1005208 (2015).
50. M. Hirano, et al., Direct interaction between nucleolin and hepatitis C virus NS5B. J Biol Chem 278, 5109–15 (2003).
51. M. Barel, K. Meibom, A. Charbit, Nucleolin, a shuttle protein promoting infection of human monocytes by Francisella tularensis. PLoS One 5, e14193 (2010).
52. L. Li, et al., PDLIM2 repression by ROS in alveolar macrophages promotes lung tumorigenesis. JCI Insight 6 (2021).
53. J. Yu, et al., PDlim2 selectively interacts with the PDZ binding motif of highly pathogenic avian H5N1 influenza A virus NS1. PLoS One 6, e19511 (2011).
54. G. J. B. Philippe, D. J. Craik, S. T. Henriques, Converting peptides into drugs targeting intracellular protein-protein interactions. Drug Discov Today 26, 1521–1531 (2021).
55. V. Azzarito, K. Long, N. S. Murphy, A. J. Wilson, Inhibition of α-helix-mediated protein-protein interactions using designed molecules. Nat Chem 5, 161–73 (2013).
56. J.-S. Lee, et al., FLIP-mediated autophagy regulation in cell death control. Nat Cell Biol 11, 1355–62 (2009).
57. A. Schuffenhauer, N. Brown, P. Selzer, P. Ertl, E. Jacoby, Relationships between Molecular Complexity, Biological Activity, and Structural Diversity. J Chem Inf Model 46, 525–35.
58. D. v Klopfenstein, et al., GOATOOLS: A Python library for Gene Ontology analyses. Sci Rep 8, 10872 (2018).
59. B. T. Sherman, et al., DAVID: a web server for functional enrichment analysis and functional annotation of gene lists (2021 update). Nucleic Acids Res (2022) https:/doi.org/10.1093/nar/gkac194
